# Expanded Potential Stem Cells from Human Embryos Have an Open Chromatin Configuration with Enhanced Trophoblast Differentiation Ability

**DOI:** 10.1002/advs.202204797

**Published:** 2023-02-12

**Authors:** Andy Chun Hang Chen, Yin Lau Lee, Hanzhang Ruan, Wen Huang, Sze Wan Fong, Siyu Tian, Kai Chuen Lee, Genie Minju Wu, Yongqi Tan, Timothy Chun Hin Wong, Jian Wu, Weiyu Zhang, Dandan Cao, Judy Fung Cheung Chow, Pengtao Liu, William Shu Biu Yeung

**Affiliations:** ^1^ Department of Obstetrics and Gynaecology, School of Clinical Medicine Li Ka Shing Faculty of Medicine The University of Hong Kong Hong Kong Hong Kong; ^2^ Shenzhen Key Laboratory of Fertility Regulation Reproductive Medicine Center The University of Hong Kong ‐ Shenzhen Hospital Shenzhen 518000 China; ^3^ Centre for Translational Stem Cell Biology Building 17 W The Hong Kong Science and Technology Park Hong Kong Hong Kong; ^4^ School of Biomedical Sciences Li Ka Shing Faculty of Medicine The University of Hong Kong Stem Cell and Regenerative Medicine Consortium Hong Kong Hong Kong

**Keywords:** chromatin remodeling, expanded potential stem cells, hippo signaling pathway, trophoblast differentiation

## Abstract

Human expanded potential stem cells (hEPSC) have been derived from human embryonic stem cells and induced pluripotent stem cells. Here direct derivation of hEPSC from human pre‐implantation embryos is reported. Like the reported hEPSC, the embryo‐derived hEPSC (hEPSC‐em) exhibit a transcriptome similar to morula, comparable differentiation potency, and high genome editing efficiency. Interestingly, the hEPSC‐em show a unique H3 lysine‐4 trimethylation (H3K4me3) open chromatin conformation; they possess a higher proportion of H3K4me3 bound broad domain (>5 kb) than the reported hEPSC, naive, and primed embryonic stem cells. The open conformation is associated with enhanced trophoblast differentiation potency with increased trophoblast gene expression upon induction of differentiation and success in derivation of trophoblast stem cells with bona fide characteristics. Hippo signaling is specifically enriched in the H3K4me3 broad domains of the hEPSC‐. Knockout of the Hippo signaling gene, YAP1 abolishes the ability of the embryo‐derived EPSC to form trophoblast stem cells.

## Graphical abstract

hEPSC‐em show enhanced trophoblast differentiation potency through a unique H3 lysine‐4 trimethylation (H3K4me3) open chromatin conformation. *YAP1* is specifically enriched in the H3K4me3 broad domains of the hEPSC‐em. *YAP1* knockout hindered trophoblast differentiation through disruption of cell polarity.

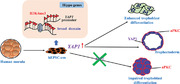

## 1 Introduction

Epigenetic modifications are highly dynamic in the course of pre‐implantation embryo development. Genome‐wide profiling of mouse^[^ ^1^ ^]^ and human^[^ ^2^ ^]^ embryos reveals correlation of histone H3 lysine 4 trimethylation (H3K4me3) and histone H3 lysine 27 trimethylation (H3K27me3) with changes in corresponding gene expression. Histone remodeling occurs during lineage segregation into inner cell mass (ICM) and trophectoderm (TE). For instance, the H3K27me3 distribution patterns in lineage‐specific genes of the TE cells and the epiblast (EPI) cells or ICM of human blastocysts are different.^[^ ^2^ ^]^ While the global H3K4me3 level at promoters is constitutively maintained throughout pre‐implantation development, majority of the TE‐specific promoters are devoid of the repressive H3K27me3 mark in human blastocysts.^[^ ^2^ ^]^ On the other hand, the porcine embryos exhibited an enhanced H3K4me3 level in the TE cells.^[^ ^3^ ^]^ Histone remodeling also occurs during trophoblast differentiation from human embryonic stem cells (hESC); majority of the bivalent marked TE‐specific transcription factors, including *GATA2*, *GATA3*, *TFAP2A*, and *TFAP2C*, become monovalent H3K4me3 marked during early trophoblast differentiation.^[^ ^4^ ^]^ It is apparent that correct histone remodeling is important for the differentiation of TE and early trophoblast.

Recently, we established the expanded potential stem cells (EPSC).^[^ ^5^ ^]^ Primed hESC and induced pluripotent stem cells (hiPSC) are converted into human expanded potential stem cells (hEPSC‐ES) through inhibition of the GSK3, tankyrases, SRC, and BRAF pathways.^[^ ^5^ ^]^ hEPSC‐ES possess transcriptomic features like that of blastomere of embryos at the eight‐cell and morula stage. They exhibit enhanced pluripotent features and can be differentiated into trophoblast lineages efficiently. Importantly, the transcriptomes of hEPSC‐ES lines are more homogeneous than the primed hESC at single‐cell level.^[^ ^5^ ^]^ Here, by modulating similar molecular pathways, we reported the establishment of hEPSC directly from human pre‐implantation embryos (hEPSC‐em) donated for research. The hEPSC‐em could be differentiated into various cell lineages including trophoblast, pancreatic cells, and germ cells. Although the hEPSC‐em and hEPSC‐ES had similar transcriptome, the hEPSC‐em exhibited a unique H3K4me3 open chromatin conformation when compared to the naive hESC and the primed hESC. Hippo signaling was specifically enriched in the H3K4me3 bound broad domains of the hEPSC‐em and contributed to enhanced trophoblast differentiation potency of the cells.

## 2 Results

### 2.1 Establishment of EPSC from Human Pre‐Implantation Embryos

**Figure 1:**
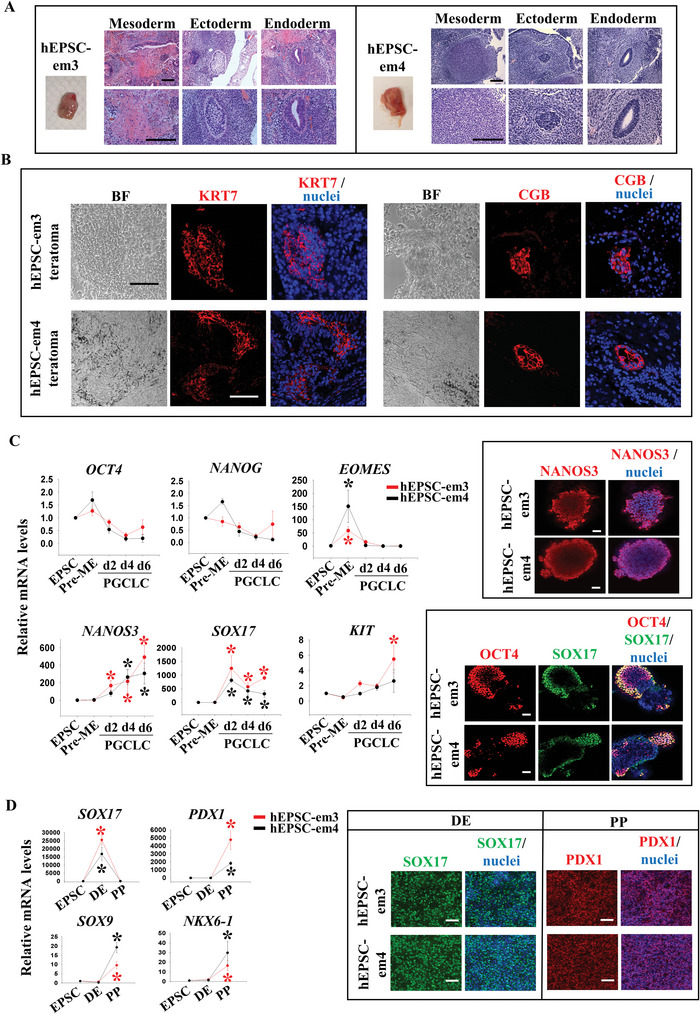
Establishment of EPSC from human pre‐implantation embryos (hEPSC‐em). A) Haematoxylin and eosin staining of teratoma sections derived from hEPSC‐em3 (left) and hEPSC‐em4 (right). Bone cartilage or striated muscle structure (mesoderm), neural tissue structure (ectoderm), and glandular epithelium structure (endoderm) were observed. Scale bar: 100 µm. B) Immunofluorescence staining of trophoblast markers (KRT7 and CGB) on teratoma sections derived from hEPSC‐em3 (top) and hEPSC‐em4 (bottom). The nuclei were stained with Hoechst 33258. Scale bar: 100 µm. C) RT‐qPCR analysis of *OCT4*, *NANOG*, *EOMES*, *NANOS3*, *SOX17*, and *KIT* during PGCLC formation from hEPSC‐em (left). *p < 0.05 compared to EPSC control, *t*‐test; *n* = 3. Immunofluorescent staining of PGC markers (NANOS3, SOX17, and OCT4) following 6 days of PGCLC induction (right). The nuclei were stained with Hoechst 33258. Scale bar: 100 µm. D) RT‐qPCR analysis of DE marker (*SOX17*) and PP markers (*PDX1*, *SOX9*, and *NKX6‐1*) during pancreatic differentiation from hEPSC‐em (left). *p < 0.05 compared to EPSC control; *t*‐test; *n* = 3. Immunofluorescence staining of pancreatic markers (SOX17 and PDX1) following pancreatic differentiation from hEPSC‐em (right). The nuclei were stained with Hoechst 33258. Scale bar: 100 µm. DE: definitive endoderm, PP: pancreatic progenitor.

We derived hEPSC‐em directly from human pre‐implantation embryos. The embryos at four‐cell stage donated for research were cultured to morulae or early blastocysts before culturing on mouse STO feeder cells in hEPSC culture medium (Figure S1A, Supporting Information).^[^ ^5^ ^]^ The attached embryos were mechanically dissected into fragments for the first few passages. Thereafter, the emerged colonies were enzymatically digested and cultured in the hEPSC medium. Out of the 116 frozen four‐cell embryos thawed for the derivation, 82 developed to morulae or early blastocysts. From these embryos, three male and two female stable hEPSC‐em lines were established. The derivation efficiency was 6%. All the five lines displayed normal karyotypes (Figure S1B, Supporting Information). Detailed characterization was performed on one female (hEPSC‐em3) and one male (hEPSC‐em4) lines. Normal karyotypes were maintained in the two lines after culture for over 40 passages (Figure S1C, Supporting Information). They expressed protein of pluripotent markers (OCT4 and NANOG) and not early differentiation marker KRT18 (Figure S1D, Supporting Information). The pluripotent characteristics of the hEPSC‐em lines were confirmed in vivo by the formation of teratoma containing mesodermal (bone cartilage), ectodermal (neural), and endodermal (glandular epithelium) structures (**Figure** **1**A). Critically, cells expressing markers of trophoblast lineages (KRT7 and CGB) were detected in some regions of the teratoma (Figure 1B).

We examined the differentiation potential of four hEPSC‐em lines (hEPSC‐em1, hEPSC‐em3, hEPSC‐em4, and hEPSC‐em5) into germline and pancreatic lineage. A published protocol for germ cells differentiation was used.^[^ ^6^ ^]^ Upon a 12 h exposure to activin A and CHIR99021, pre‐mesendoderm cells (pre‐ME) with enhanced *EOMES* expression (Left panel, Figure 1C and Figure S1E, Supporting Information) were induced. The pre‐ME were further differentiated into primordial germ cell‐like cells (PGCLCs) in embryoid bodies (EB, Figure 1C and Figure S1F, Supporting Information); the mRNA expression of typical PGCLC markers (*NANOS3, SOX17*, and *KIT*) were significantly induced after 6 days of differentiation. In addition, PGCLC also retained certain levels of pluripotent marker expressions including *OCT4* and *NANOG* (Left panel, Figure 1C and Figure S1F, Supporting Information). The derived PGCLC in EB were NANOS3^+^ (Top right panel, Figure 1C). More importantly, a portion of the cells in the EB co‐expressed SOX17 and OCT4 (Bottom right panel, Figure 1C), a hallmark feature of PGCLC.^[^ ^6^, ^7^ ^]^

The hEPSC‐em were also differentiated into definitive endoderm (DE) and pancreatic progenitor (PP) cells as reported.^[^ ^8^ ^]^ The differentiation was efficient with the differentiated cells expressing *SOX17* at the DE stage and *PDX1, SOX9*, and *NKX6‐1* at the PP stage (Left panel, Figure 1D and Figure S1G, Supporting Information). Immunofluorescence staining indicated that >95% of the cells at the DE and the PP stages were SOX17^+^ and PDX1^+^, respectively (Right panel, Figure 1D).

### 2.2 Efficient Trophoblast Differentiation from hEPSC‐em

**Figure 2:**
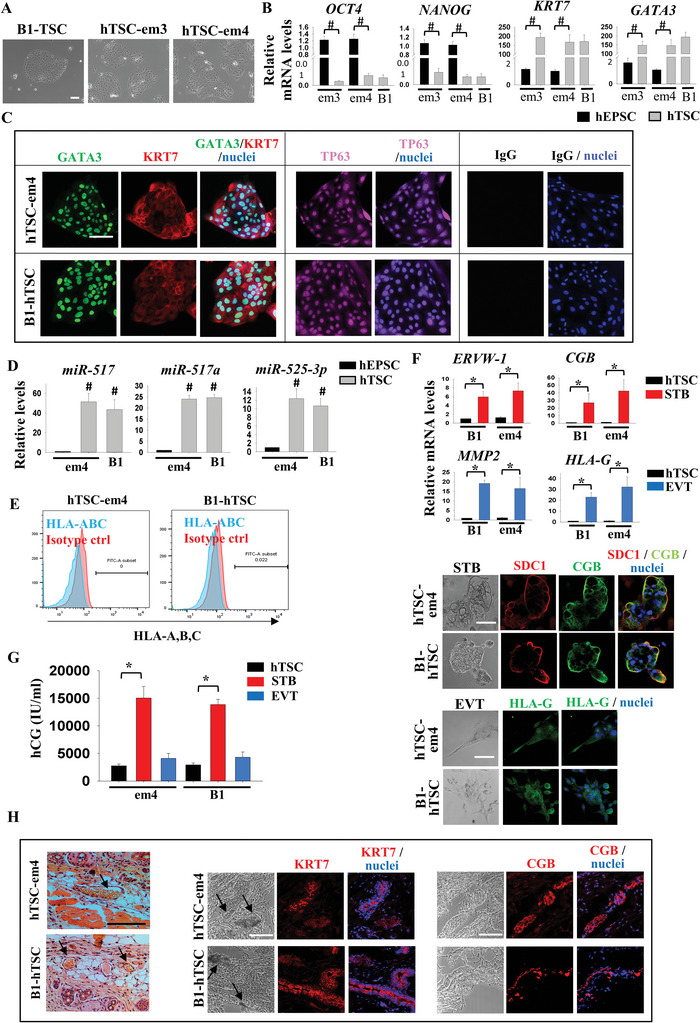
Efficient trophoblast differentiation from hEPSC‐em. A) Images of human trophoblast stem cell (hTSC) derived from hEPSC‐em (hTSC‐em3 and hTSC‐em4), and hTSC derived from human blastocyst (B1‐TSC). Scale bar: 100 µm. B) RT‐qPCR analysis of pluripotent markers (*OCT4* and *NANOG*) and trophoblast markers (*CDX2*, *KRT7*, and *GATA3*) in hTSC‐em (grey bars) #*p* < 0.001 compared to EPSC control (black bars). B1‐TSC was included as control; *t*‐test; *n* = 3. C) Immunofluorescence staining of KRT7, GATA3, and TP63 in hTSC‐em4 and B1‐hTSC. The nuclei were stained with Hoechst 33258. Scale bar: 100 µm. D) RT‐qPCR analysis of C19MC miRNAs (miR‐517, miR‐517a, and miR‐525‐3p) in hTSC‐em. #*p* < 0.001 compared to EPSC control (black bars). B1‐hTSC was included as control; *t*‐test; *n* = 4. E) Flow cytometry analysis of HLA‐A, ‐B, and ‐C expression in hTSC‐em and B1‐TSC. F) RT‐qPCR analysis of *ERVW‐1* and *HCG* in syncytiotrophoblast (STB, red bars), and *MMP2* and *HLA‐G* in extravillous trophoblast (EVT, blue bars) differentiated from hTSC of hEPS‐em4 and B1 (black bars). *p < 0.05 compared to hTSC control; *t*‐test; *n* = 3 (top). Immunofluorescence staining of SDC1, CGB, and HLA‐G in hTSC‐em4 and B1‐hTSC differentiated cells. The nuclei were stained with Hoechst 33258. Scale bar: 100 µm (bottom). G) hCG secretion level (IU mL^−1^) in spend media of hTSC‐em4 and B1‐hTSC differentiated cells. *p < 0.05 compared to hTSC control. *n* = 3. H) Haematoxylin and eosin staining of skin sections upon subcutaneous injection of hTSC‐em4 and B1‐hTSC. Black arrow indicated the blood‐filled lacunae (left). Immunofluorescence staining of KRT7 and CGB of the sections. Black arrow indicated the blood‐filled lacunae. The nuclei were stained with Hoechst 33258. Scale bar: 100 µm (right).

To study trophoblast differentiation of the hEPSC‐em, we converted hEPSC‐em into human trophoblast stem cells (hTSC) using a published protocol.^[^ ^9^ ^]^ After five passages, TSC‐like colonies emerged and stable hTSC lines were formed from both hEPSC‐em lines (hTSC‐em). The morphologies of the hTSC‐em colonies resembled those derived from human blastocyst (B1‐TSC)^[^ ^9^ ^]^ (**Figure** **2**A). As expected, the hTSC‐em expressed low level of the pluripotency markers *OCT4* and *NANOG* but high levels of the trophoblast markers *KRT7* and *GATA3* (Figure 2B). The transcript levels of *KRT7* and *GATA3* in the hTSC‐em were comparable to that in the B1‐TSC. Immunofluorescence staining confirmed protein expression of KRT7, GATA3, and TP63 in the hTSC‐em and B1‐TSC (Figure 2C). In addition, the chromosome 19 microRNA cluster (C19MC), miR‐517, miR‐517a, and miR‐525‐3p were highly expressed in the hTSC‐em and B1‐TSC when compared to the hEPSC‐em (Figure 2D). Both the hTSC‐em and the B1‐TSC did not express HLA‐A, ‐B, and ‐C (Figure 2E). The hTSC‐em could be differentiated into syncytiotrophoblast (STB) by treatment with forskolin, and into extravillous trophoblast (EVT) by NRG‐1 and A83‐01 as reported.^[^ ^9^ ^]^ Upon induction of differentiation, the gene expression levels of STB (*ERVW‐1* and *CGB*) and EVT (*MMP2* and *HLA‐G*) markers were comparable to that of the B1‐TSC. Immunoreactivities of SDC1 and CGB were detected in the STB‐like cells, and that of HLA‐G was detected in the EVT‐like cells upon differentiation in both hTSC (Figure 2F). We measured the production of HCG by the hTSC and the differentiated cells using ELISA. Significantly higher levels of HCG was detected in the spent medium of the STB‐like cells when compared to that of the hTSC and EVT‐like cells (Figure 2G). To assess the in vivo differentiation potential of hTSC‐em, we injected hTSC‐em and B1‐TSC subcutaneously into NOD‐SCID mice. Similar to B1‐TSC^[^ ^9^ ^]^ and our previous study,^[^ ^5^ ^]^ the injected cells formed lesions by day 7 and invaded the dermal and subcutaneous tissues as demonstrated by the presence of KRT7 and CGB positive cells, and blood‐filled lacunae in the lesions (Figure 2H). The results demonstrated efficient differentiation of the hEPSC‐em into trophoblast lineage through hTSC formation.

To examine genome editing efficiency in hEPSC‐em, we generated reporter cell lines for the trophoblast transcription factor *GATA3*. Knock‐in of a T2A‐mCherry cassette into the last exon of the *GATA3* locus was performed in the hEPSC‐em using the CRISPR‐Cas9 approach (Figure S1H, Supporting Information). After electroporation of the Cas‐9 guide RNAs (gRNAs) and the donor vectors into the hEPSC‐em, the transfected cells were fluorescence‐activated cell sorting (FACS) sorted for green fluorescent protein, which was co‐expressed with the Cas9 protein. 14 clones from each line were chosen after antibiotic selection. PCR analyses revealed that all of them had the mCherry cassette correctly inserted. Among them, one hEPSC‐em4 clone (clone 13) possessed homozygous knock‐in of the reporter cassette (Figure S1H, Supporting Information). DNA sequencing of the 5′ and 3′ junction PCR products indicated that the mCherry cassette was inserted precisely before the *GATA3* stop codon at the last exon (data not shown). The results demonstrated a high genome editing efficiency in the hEPSC‐em as compared to the reported knock‐in efficiency of 8–60% in the primed hESC.^[^ ^10^, ^11^ ^]^ Upon induction of trophoblast differentiation through hTSC formation, the mCherry fluorescent signal was detected in the *GATA3*‐mCherry hEPSC‐em4 cells (Figure S1I, Supporting Information).

### 2.3 hEPSC‐em Lines Are Transcriptionally Similar to Human Morula

**Figure 3:**
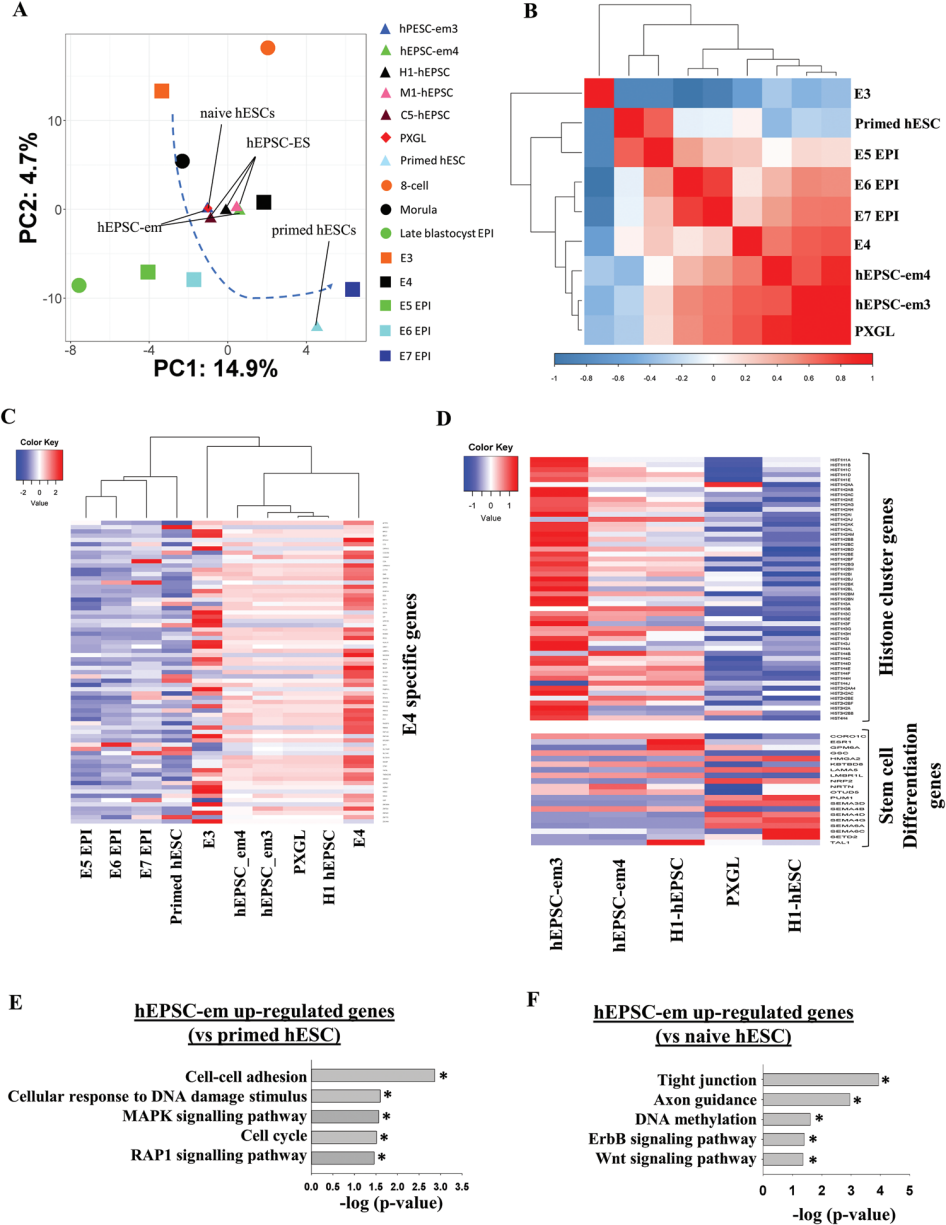
Single cell RNA sequencing analysis of hEPSC‐em transcriptomes. A) PCA of scRNA‐Seq data of hEPSC‐em3, hEPSC‐em4, hEPSC‐ES, naive hESC, primed hESC, and human pre‐implantation embryos. scRNA‐seq data of hEPSC‐ES were from ref. [5], and that of the human pre‐implantation embryos, naive, and primed hESC were from refs. [12, 13] and [14], respectively. hEPSC‐em3, *n* = 3051; hEPSC‐em‐4, *n* = 3170; hEPSC‐ES, *n* = 96; naive hESC, *n* = 100; primed hESC, *n* = 26; eight‐cell, *n* = 20; morula, *n* = 16; EPI, *n* = 5; E3, *n* = 81; E4, *n* = 190; E5 EPI, *n* = 41; E6 EPI, *n* = 45; E7 EPI, *n* = 41. *n* represents the number of cells. The average positions of each cell types were labeled in the PCA plot. B) Pearson correlation analysis of the gene expression patterns of hEPSC‐em3, hEPSC‐em4, hEPSC‐ES, primed hESC, and naive hESC. The correlation matrix was clustered using Pearson's correlation. C) Heatmap clustering showing the expression values of E4 specific genes among hEPSC‐em3, hEPSC‐em4, hEPSC‐ES, naive hESC, primed hESC, and human pre‐implantation embryos. D) The expression patterns of histone cluster genes (top) and stem cell differentiation genes (bottom) between hEPSC‐em, hEPSC‐ES, primed hESC, and naive hESC. The expression values were normalized average counts of single‐cell transcriptome patterns among different cell liens. E) Gene ontology analysis of the hEPSC‐em upregulated genes as compared to the primed hESC. *p < 0.05. F) Gene ontology analysis of the hEPSC‐em upregulated genes as compared to the primed hESC. *p < 0.05.

Single‐cell RNA‐seq (scRNA‐Seq) was performed on the two hEPSC‐em lines. Their transcriptomes were compared to two reported profiles of human pre‐implantation embryos,^[^ ^12^, ^13^ ^]^ hEPSC,^[^ ^5^ ^]^ naive hESCs (PXGL),^[^ ^14^ ^]^ and primed hESC.^[^ ^12^ ^]^ Principal component analysis (PCA) showed that the hEPSC‐em were transcriptionally more similar to blastomeres on embryonic day 4 (E4) than blastomeres of other pre‐implantation stages. In addition, hEPSC (hEPSC‐em3, ‐em4, H1‐, C5‐, and M1‐hEPSC) and naive hESCs were clustered closely together in the PCA plot. On the other hand, the transcriptome of the primed hESC (H1‐hESC)^[^ ^12^ ^]^ was distinct from that of the hEPSC‐em but similar to that of the E7 EPI (**Figure** **3**A). Pearson correlation analysis of the top 500 variable genes showed that the transcriptome of the hEPSC was distinct from that of the primed hESC, but highly correlated with that of the naive hESC^[^ ^14^ ^]^ (Figure 3B). Similarly, the primed hESC was highly correlated with the pre‐implantation epiblast (E5 EPI); while the hEPSC and the naive hESC were highly correlated with the blastomeres at E4 (Figure 3B). We further identified the morula specific genes relative to other developmental stages of the human pre‐implantation embryos^[^ ^13^ ^]^ (Figure 3C). All analyses indicated close clustering of the naive hESC with the hEPSC‐ES, hEPSC‐em, and morula (E4) but distinct from the primed hESCs and the epiblast (E5–E7). On the contrary, the epiblast specific genes relative to other embryonic stages^[^ ^13^ ^]^ showed high induction levels in the primed hESCs than in the naive hESC and the hEPSCs (Figure S2A, Supporting Information). We have previously shown that the hEPSC converted from hESC or hiPSC (hEPSC‐ES; H1‐ and C5‐hEPSC) had high expression of histone genes (*HIST1H1C*, *HIST1H2AC*, *HIST1H2BD*, and *HIST1H2BJ*).^[^ ^5^ ^]^ Here, we further demonstrated that most of the histone cluster genes (e.g., *HIST1H1C* and *HIST1H2BD*) were highly expressed in both the hEPSC‐em lines and the hEPSC‐ES when compared to the naive hESC and the primed hESC. In addition, the lineage‐related differentiation genes (e.g., *NRP2* and *LAMA5*) were expressed at lower levels in the hEPSC‐em and the hEPSC‐ES than the other two stem cell types (Figure 3D). In addition, the expression values of *HIST1H1C* and *HIST1H2BD* were found to be higher in hEPSC‐em and hEPSC‐ES when compared to naive and primed hESCs; while *NRP2* and *LAMA5* were found to be lower in hEPSCs when compared to naive and primed hESCs (Figure S2B, Supporting Information). We selected two histone cluster genes *HIST1H1C* and *HIST1H2BD* for quantitative polymerase chain reaction (qPCR) analysis, and validated that their expression were significantly higher in hEPSCs than in primed hESC (Figure S2B, Supporting Information).

Gene ontology analysis of the transcriptomes between the hEPSC‐em and the primed hESC showed that the genes significantly upregulated in the hEPSC‐em were enriched in biological processes including cell–cell adhesion, cellular response to DNA damage, MAPK signaling pathway, cell cycle, and RAP1 signaling pathway (Figure 3E). Among the eight upregulated genes involved in cellular response to DNA damage, six were highly expressed in the eight‐cell or the morula stages (Figure S2C, Supporting Information). The expression of DNA repair genes, *GNL1*, *SGK1*, *ZBTB1*, *TLK2*, *RASSF1*, and *XIAP* were significantly higher in the hEPSC‐em than the primed hESC (Figure S2D, Supporting Information).

Similar comparison between the hEPSC‐em and the naive hESC showed enrichment of terms including tight junction, axon guidance, DNA methylation, ErbB, and Wnt signaling pathways in the hEPSC‐em (Figure 3F). The hEPSC‐em also expressed higher levels of the DNA repair genes *GNL1* and *TLK2* than the naive hESC (Figure S2E, Supporting Information).

We next compared the transcriptomes between hEPSC‐em and hEPSC‐ES. Despite high similarity in the transcriptomes, minor differences were noted between them. We subjected their differentially expressed genes to clustering analysis with respect to those in human pre‐implantation embryos. Interestingly, among the genes significantly upregulated in the hEPSC‐em, a large proportion of them (69%) were enriched in embryos from the zygotic to morula stages (Figure S2F, Supporting Information). On the other hand, around half (50%) of genes significantly upregulated in the hEPSC‐ES were enriched in EPI and TE of blastocysts (Figure S2F, Supporting Information). For instance, *KRT8*, a TE specific gene,^[^ ^13^ ^]^ was significantly upregulated in the hEPSC‐ES compared with the hEPSC‐em (Figure S2G, Supporting Information). Three of the hEPSC‐em upregulated genes (*NPM3*, *FERMT1*, and *MTHFD2L*) (Figure S2H, Supporting Information) were important for pluripotency maintenance in mouse ESCs^[^ ^15^, ^16^, ^17^ ^]^ though their roles in humans remained to be explored.

### 2.4 hEPSC‐em Exhibit a Unique Broad H3K4me3 Conformation

We determined the H3K4me3 chromatin conformation of the hEPSC‐em lines by chromatin immunoprecipitation sequencing (ChIP‐seq), and compared to published datasets on naive hESC, primed hESC,^[^ ^18^ ^]^ and hEPSC‐ES (C5 and H1 hEPSC).^[^ ^5^ ^]^ The overall H3K4me3 levels were similar among the stem cell lines (Figure S3A, Supporting Information). In the hEPSC‐em, the gene promoters bound by H3K4me3 alone corresponded to open chromatin structure mainly enriched in terms of stem cell maintenance, cell cycle regulation, and histone methylation (Figure S3B, Supporting Information).

**Figure 4:**
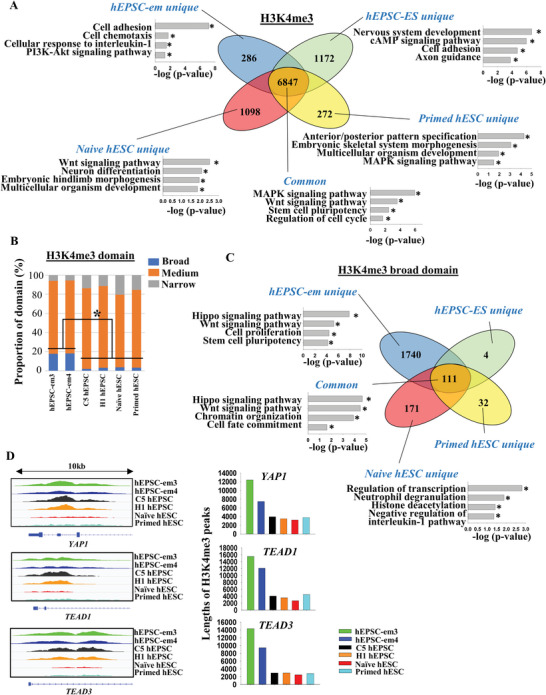
hEPSC‐em had unique broad H3K4me3 conformation. A) ChIP‐seq analysis comparing the number of H3K4me3‐marked gene promoters between the hEPSC‐em, hEPSC‐ES, primed, and naive hESC. Venn diagram indicated the number of gene promoters marked by each cell line. Gene ontology analysis for common and unique H3K4me3‐marked gene promoters of each cell line was displayed. The ChIP‐seq data of naive and primed hESC was from ref. [18]. B) Proportions (%) of broad (>5 kb, blue bars), medium (1–5 kb, orange bars), and narrow (<1 kb, grey bars) H3K4me3 domains between cell lines. *p < 0.05 comparing different cell lines, *t*‐test. C) ChIP‐seq analysis comparing the number of broad H3K4me3 domain between hEPSC‐em, hEPSC‐ES, primed, and naive hESC. Venn diagram indicated the number of gene promoters marked by each cell line. Gene ontology analysis for common and unique H3K4me3 broad domain of each cell line was displayed. D) The lengths of H3K4me3 peaks in *YAP1*, *TEAD1*, *TEAD3* between different cell lines were shown (left). The tracks showing H3K4me3 peak at the gene loci (*YAP1*, *TEAD1*, and *TEAD3*) in hEPSC‐em, hEPSC‐ES, primed hESC, and naive hESC (right).

We next compared the H3K4me3‐bound promoters in hEPSC‐em with those in hEPSC‐ES, naive hESC, and primed hESC. Interestingly, majority of the H3K4me3‐bound promoter genes (85–95%) were shared in all the stem cells studied. Gene ontology analysis revealed that the common genes were enriched in MAPK and Wnt signaling pathways, stem cell pluripotency, and regulation of cell cycle (**Figure** **4**A). On the other hand, most of the H3K4me3‐bound genes specific to the stem cells studied were not related to stem cell maintenance or related characteristics (Figure 4A). Consistent with our findings on the hEPSC‐ES,^[^ ^5^ ^]^ the promoters of pluripotent markers (*OCT4*, *NANOG*, and *SOX2*) were marked by the active H3K4me3 in the hEPSC‐em. The observation was similar across different stem cell lines, with the hEPSC generally showing higher H3K4me3 enrichment in the promoters than that of the naive hESC and the primed hESC (Figure S3C, Supporting Information). In addition, the H3K4me3 of germ‐cell related gene (*TFAP2C* and *KIT*) promoters were more enriched in the hEPSC than the naive hESC and the primed hESC (Figure S3C, Supporting Information), in line with the observed potency of the hEPSC‐em in germ cell lineage differentiation.

The peaks of the H3K4me3‐bound promoters were classified into broad (>5 kb), medium (1–5 kb), and narrow (<1 kb) domains for further analyses. Genes bound with the histone marks in the broad domains are highly associated with their expression levels.^[^ ^1^, ^19^ ^]^ Consistently, genes with the board H3K4me3 domains had significantly higher expression than those with the narrow domains in the hEPSC‐em and the hEPSC‐ES (Figure S3D, Supporting Information). Interestingly, the proportion of the broad H3K4me3 peaks in the hEPSC‐em (≈15%) was significantly higher than that in the hEPSC‐ES, naive hESC, and primed hESC (≈1–3%) (Figure 4B).

The gene promoters having the H3K4me3 broad domains between the hEPSC‐em (1851), hEPSC‐ES (115), naive hESC (282), and primed hESC (143) were compared (Figure 4C). The hEPSC‐em specific H3K4me3 broad domains were enriched for Hippo and Wnt signaling pathway, cell proliferation, and stem cell pluripotency. Those specific to the naive hESC were enriched for regulation of transcription, neutrophil degranulation, histone deacetylation, and negative regulation of interleukin‐1 pathway. The number of genes specific to the hEPSC‐ES^[^ ^4^ ^]^ and the primed hESC^[^ ^32^ ^]^ were too few to have significant enrichment. For the H3K4me3 broad domain bound gene promoters common in all the stem cell types, Hippo and Wnt signaling pathway, chromatin organization, and cell fate commitment were enriched. It was noted that some genes of the Hippo signaling pathways with the H3K4me3 broad domain including *YAP1*, *TEAD1*, and *TEAD3*, were specifically enriched in the hEPSC‐em, while others such as *GSK3B*, *TCF7L2*, and *FZD5* are common in all the stem cell studied (Figure 4C and Figure S3E, Supporting Information). It was noted that the peak lengths of Hippo‐related genes in the hEPSC‐em were longest among the stem cell types studied (Figure 4D). Consistent with the broad H3K4me3 domain, we found higher basal YAP1 expression in hEPSC‐em than the other pluripotent cell types (Figure S3F, Supporting Information). Interestingly, upon culture of the hEPSC‐em for over 50 passages, we noticed similar expression of Hippo (*YAP1*) and Wnt (*WNT3A*) related genes that were bound by the H3K4me3 broad domains between the early^[^ ^20^, ^21^, ^22^, ^23^, ^24^, ^25^, ^26^, ^27^, ^28^, ^29^, ^30^, ^31^, ^32^, ^33^, ^34^, ^35^, ^36^, ^37^, ^38^, ^39^, ^40^ ^]^ and late (>50) passaged hEPSC‐em (Figure S3G, Supporting Information). These genes also exhibited similar expression patterns upon BAP induction. The results suggested that the broad H3K4me3 could be maintained at least up to 50 passages.

### 2.5 hEPSC‐em Are Susceptible to Induction of Trophoblast Differentiation

The hEPSC‐em and the hEPSC‐ES could form hTSC efficiently. Since the conversion of hEPSC into hTSC using the reported method^[^ ^9^ ^]^ was a gradual process that took approximately five passages, we sought the BAP trophoblast differentiation protocol by simultaneous activation of BMP4 and inhibition of FGF and TGF*β* signaling^[^ ^5^ ^]^ to capture the TE and the early trophoblast states from hEPSC.

#### 2.5.1 BAP Treatment Induced Mainly Trophoblastic but Not Amniotic Cells Differentiation from hEPSC

There are recent debates on differentiation of primed hESC and hEPSC‐ES into amnion epithelial cells (AME) or trophoblast cells upon BMP stimulation.^[^ ^14^, ^20^ ^]^ The only available dataset for capturing early human AME development was from post‐implantation human embryos up to d14.^[^ ^21^ ^]^ However, the dataset was being questioned for misclassification of AME cells due to inclusion of pseudogenes.^[^ ^22^, ^23^ ^]^ Here, we selected the AME cells of post‐implantation human embryos^[^ ^21^ ^]^ after re‐annotation of the cells as reported recently,^[^ ^22^ ^]^ and identified the AME specific genes relatively to other cell lineages in post‐implantation human embryo dataset. These AME specific genes were highly expressed in the EPI but not TE of E6 and E7 pre‐implantation human embryos^[^ ^13^ ^]^ (Figure S4A, Supporting Information). Their expression in H1‐ and C5‐hEPSC post‐BAP treatment was examined. Heatmap of the differentiating samples showed induction of expression of the TE‐ and cytotrophoblast (CTB)‐specific genes^[^ ^13^, ^21^ ^]^ from d4 onwards, and the STB‐ and EVT‐specific genes from d6 onwards (Figure S4B, Supporting Information). The AME‐specific genes did not show a specific expression pattern upon BAP‐treatment (Figure S4B, Supporting Information). We also compared the data with that of cynomolgus‐AME^[^ ^24^ ^]^ after cell re‐annotation,^[^ ^22^ ^]^ and found induction of a subset of cynomolgus‐AME genes only during late stage (from d9 onwards) of hEPSC‐ES differentiation (Figure S4C, Supporting Information).

The BAP trophoblastic spheroid model (BAP‐EB) was applied to hEPSC‐em. Similar to primed hESC,^[^ ^25^, ^26^ ^]^ BAP‐EB from the hEPSC‐em exhibited a cystic structure with size and morphology resembled that of human blastocysts (Figure S5A, Supporting Information), and expressed early trophoblast markers (CDX2 and GATA3) at 48 h post‐differentiation and late trophoblast markers (HCG) at later time points (Figure S5B, Supporting Information). BAP‐EB of hEPSC‐em from 72 h post‐differentiation selectively attached onto the receptive endometrial epithelial cells (Ishikawa cells) but not the non‐receptive endometrial epithelial cells (HEC1B cells) (Figure S5C, Supporting Information). We further performed scRNA‐seq on BAP‐EB derived from hEPSC‐em4 at 48, 72, and 96 h post‐differentiation. The results demonstrated that the TE markers (*HAND1*, *EPCAM*, and *GATA3*) were highly induced at all the three timepoints, but the AME markers (*HOXA11*, *SNAI1*, and *TNC*) were barely detected (Figure S5D, Supporting Information), confirming that the BAP protocol did not drive AME differentiation of hEPSCs significantly in the early induction period.

We generated trophospheres^[^ ^27^ ^]^ from hTSC‐em in the TSC medium.^[^ ^9^ ^]^ Unlike BAP‐EB, the trophospheres were morphologically dissimilar to human blastocysts (Figure S6A, Supporting Information), did not attach onto the receptive endometrial cells (Figure S6B, Supporting Information). The trophospheres expressed trophoblast markers (*CDX2*, *GATA3*, and *KRT7*) that did not change significantly upon exposure to BAP for 5 days (Figure S6C, Supporting Information). On the other hand, the EVT (*MMP2* and *HLA‐G*) markers were downregulated while the STB (*ERVW‐1* and *CGB*) markers were induced with BAP treatment (Figure S6C, Supporting Information). We reasoned that the differences between BAP‐EB and BAP treated trophospheres were because BAP‐EB reflected a differentiation toward TE and trophoblast^[^ ^26^ ^]^ while trophospheres reflected a response of CTB to BAP.^[^ ^28^ ^]^ Therefore, the BAP‐EB model was used subsequently for studying early TE/trophoblast development.

#### 2.5.2 Comparison with Primed hESC

**Figure 5:**
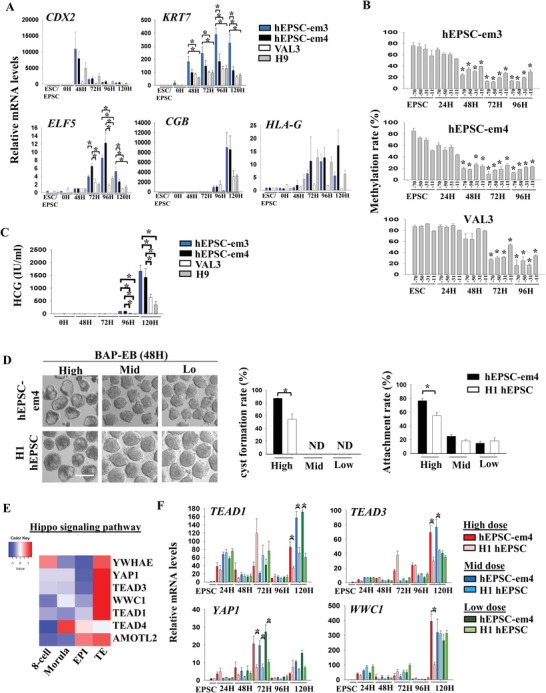
hEPSC‐em are susceptible to induction of trophoblast differentiation. A) RT‐qPCR analysis of *CDX2*, *KRT7*, *ELF5*, *HCG*, and *HLA‐G* gene expressions in BAP‐EB differentiated from hEPSC‐em‐3 (blue bars), hEPSC‐em‐4 (black bars), and primed hESC (VAL3 [white bars] and H9 [grey bars]). *p < 0.05 comparing between experimental groups; *t*‐test; *n* = 3. B) DNA methylation rates (%) of CpG sites at *ELF5* promoter during BAP‐EB differentiation in different cell lines. *p < 0.05 compared to EPSC control; *t*‐test; n = 3. C) HCG level (IU mL^−1^) in spent media collected during BAP‐EB differentiation from different cell lines. *p < 0.05 comparing EPSC control; *t*‐test; *n* = 3. D) Left: Photos showing trophoblast spheroids (BAP‐EB) differentiated from hEPSC‐em‐4 and hEPSC‐ES at 48 h under different doses of BAP. Scale bar: 100 µm. Right: proportion of cystic BAP‐EB formation and the endometrial Ishikawa cell line attachment rates between hEPSC‐em‐4 (black bars) and hEPSC‐ES (white bars) under different doses of BAP treatments. *p < 0.05 comparing between experimental groups; *t*‐test; *n* = 3. ND: not detected. E) Heatmap showing the expression levels of genes under the category of Hippo signaling pathway in human pre‐implantation embryos. scRNA‐seq data of human pre‐implantation embryos was from ref. [12]. F) RT‐qPCR analysis of *TEAD1*, *TEAD3*, *YAP1*, and *WWC1* gene expression during trophoblast differentiation from hEPSC‐em‐4 and hEPSC‐ES at different time points under high, mid, and low doses of BAP. *p < 0.05 comparing between experimental groups; *t*‐test; *n* = 3.

We compared trophoblast differentiation potency between the hEPSC‐em3, ‐em4, and the primed hESC (VAL3 and H9). The hEPSC‐em derived BAP‐EB showed significantly higher expression of the trophoblastic genes *KRT7* and *ELF5* than those from the primed hESC (**Figure** **5**A). Similar observations were obtained in hEPSC‐em1 and ‐em5 (Figure S7A, Supporting Information). *ELF5* is a gatekeeper of trophoblast differentiation and DNA demethylation of *ELF5* promoter is a hallmark of trophoblast differentiation.^[^ ^29^ ^]^ The hEPSC‐em derived trophoblastic cells exhibited faster and more extensive demethylation of the *ELF5* promoter than those from the primed hESC (Figure 5B). Consistently, the differentiated hEPSC‐em expressed significantly higher level of *ELF5* (Figure 5A) and secreted more hCG at 96 and 120 h post‐differentiation (Figure 5C) than the differentiated primed hESC.

#### 2.5.3 Comparison with hEPSC‐ES

Human TE expresses BMP receptors^[^ ^30^ ^]^ and BMP signaling is required for development of TE and extra‐embryonic lineages in mice.^[^ ^31^ ^]^ We postulated that the hEPSC‐em were more sensitive to BAP‐induced trophoblast differentiation than the hEPSC‐ES, and that a reduced induction ability in the BAP protocol could be sufficient for the induction. We first used an iPSC‐derived hEPSC line carrying a *GATA2*‐Venus reporter^[^ ^5^ ^]^ to optimize the conditions for induction of trophoblast differentiation. With unchanged concentrations of A83‐01 and PD173074, production of the Venus signal was reduced by around 40% of the control when the BMP4 concentration was reduced from 10 to 0.5 ng mL^−1^ (Figure S7B, Supporting Information). The Venus signal was maintained when the concentration of A83‐01 was reduced to 1/10 but was drastically reduced with lowering of the PD173074 concentration from 0.1 to 0.01 µm (Figure S7C, Supporting Information). Therefore, we compared trophoblast differentiation of hEPSC‐em and hEPSC‐ES in three conditions with high (BMP4: 10 ng mL^−1^, A83‐01: 1 µm, PD173074: 0.1 µm), mid (BMP4: 0.5 ng mL^−1^, A83‐01: 0.5 µm, PD173074: 0.1 µm), and low (BMP4: 0.5 ng mL^−1^, A83‐01: 0.1 µm, PD173074: 0.1 µm) induction ability of *GATA2* expression.

Despite reduced BAP doses lowering the expression of trophoblast markers (Figure S7D, Supporting Information), the expression of *CDX2*, *GATA3*, *ERVW‐1*, and *HCG* were always significantly higher in the hEPSC‐em than the hEPSC‐ES in the three tested conditions. At high BMP4 concentration, the expression of EVT markers *MMP2* and *HLA‐G* were comparable in the two types of hEPSC. However, their expression was significantly higher in the hEPSC‐em than the hEPSC‐ES cells with the low and mid induction conditions. Flow cytometry analysis also revealed significantly higher proportion of KRT7^+^ and GATA3^+^ cells at 48 and 72 h post‐differentiation in the hEPSC‐em than the hEPSC‐ES with the mid and the low induction conditions (Figure S7E, Supporting Information). In addition, only the standard high BAP dosage induced cyst formation in spheroids at 48 h post‐differentiation, when ≈80% of the treated hEPSC‐em formed cysts, which was significantly higher than that of the hEPSC‐ES (≈50%). Moreover, the spheroids derived from the hEPSC‐em had significant higher attachment rates (≈80%) on receptive endometrial epithelial cells than those from the hEPSC‐ES (≈60%) (Figure 5D). These results showed that the hEPSC‐em had higher trophoblast differentiation potency than the hEPSC‐ES.

Hippo signaling molecules (*TEAD4, YWHAE*, *YAP1*, *TEAD3*, *WWC1*, *TEAD1*, and *AMOTL2*) were enriched in the hEPSC‐em specific H3K4me3 broad domains (Figure S3E, Supporting Information). Except for *TEAD4*, the other Hippo‐related genes were highly expressed in the TE of blastocysts^[^ ^13^ ^]^ (Figure 5E). We hypothesized that the H3K4me3 bound broad domain of Hippo signaling molecules enhanced trophoblast differentiation from hEPSC‐em. Consistently, BAP treatments induced the expression of *TEAD1*, *TEAD3*, *YAP1*, and *WWC1*; the expression of *YAP1* at 72 h post‐treatment in all the doses tested, that of *TEAD1* and *TEAD3* at 120 h with the high and mid doses and that of *WWC1* at 120 h with the high dose were significantly higher in the hEPSC‐em than the hEPSC‐ES (Figure 5F). We further analyzed the common H3K4me3 broad domains bound Hippo‐related genes (*CCND1*) and found no difference in expression between the hEPSC‐em and the hEPSC‐ES upon trophoblast differentiation (Figure S7F, Supporting Information). We also quantified the expressions of the hEPSC‐em unique (*WNT3A*) and common (*FDZ5*) H3K4me3 broad domains of the Wnt molecules. It was found that significantly higher induction of *WNT3A* but not *FDZ5* was detected in the hEPSC‐em when compared to the hEPSC‐ES upon trophoblast differentiation for 96 and 120 h (Figure S7F, Supporting Information).

### 2.6 YAP1 Is Involved in Enhanced Trophoblast Differentiation in hEPSC‐em

**Figure 6:**
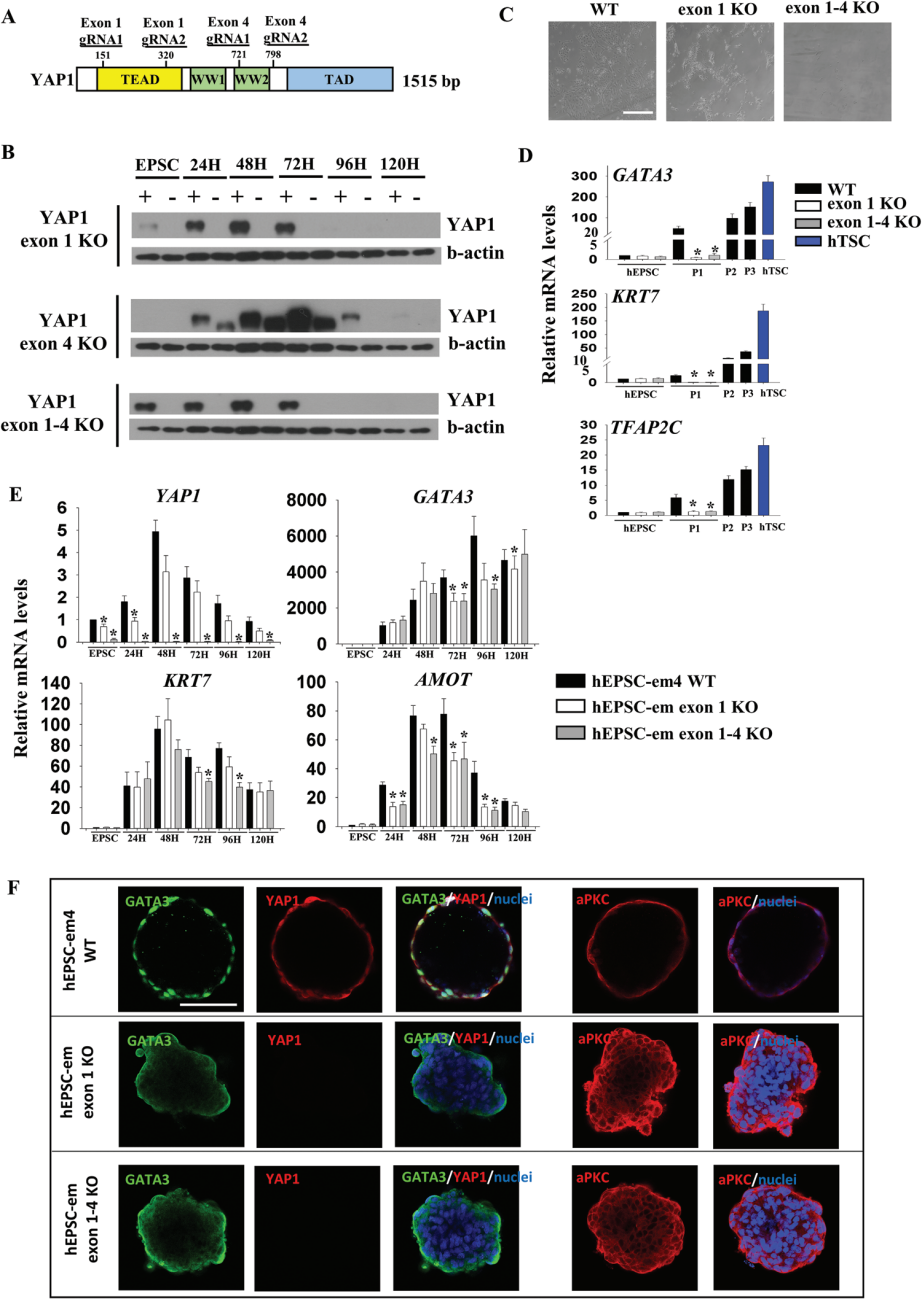
Roles of Hippo signaling molecule during trophoblast differentiation in hEPSC‐em. A) Schematic diagram showing different domains of YAP1 protein and positions of guide RNA (gRNA) for targeting *YAP1* knockout (top). B) Western blotting analyses of YAP1 protein level during trophoblast differentiation from hEPSC‐em‐4 wild‐type and *YAP1* knockout cells at exon 1, exon 4, and from exon 1–4 respectively (bottom). C) Photos showing hTSC derivation from hEPSC‐em‐4 wild‐type and *YAP1* knockout cells at exon 1 and from exon 1–4. Scale bar: 100 µm. D) RT‐qPCR analysis of *GATA3*, *KRT7*, and *TFAP2C* for hTSC derivation from hEPSC‐em‐4 wild‐type and *YAP1* knockout cells at exon 1 and from exon 1–4. *p < 0.05 comparing with wild‐type; *t*‐test; *n* = 4. ND: not detected. E) RT‐qPCR analysis of *YAP1*, *GATA3*, *KRT7*, and *AMOT* for trophoblast differentiation from hEPSC‐em‐4 wild‐type and *YAP1* knockout cells at exon 1 and from exon 1–4. *p < 0.05 comparing with wild‐type; *t*‐test; *n* = 5. F) Immunofluorescent staining of YAP1, GATA3, and aPKC in 48 h BAP‐EB derived from hEPSC‐em‐4 wild‐type and *YAP1* knockout cells at exon 1 and from exon 1–4. The nuclei were stained with Hoechst 33258. Scale bar: 100 µm.

To study the role of *YAP1* in trophoblast differentiation, we mutated *YAP1* in hEPSC‐em4 with a CRISPR/Cas9 approach. Human YAP1 protein consisted of four functional domains, namely TEAD‐binding domain (TEAD), two WW domains (WW1 and WW2), and a transactivation domain^[^ ^32^ ^]^ (schematic **Figure** **6**A). gRNAs were designed to target a 169 bp fragment of exon 1 of *YAP1*, causing ablation of the TEAD domain and mutation of the subsequent domains. Another pair of gRNAs was targeted at a 77 bp fragment of exon 4 leaving the TEAD domain intact. In addition, gRNAs from exon 1 and exon 4 were combined (excision of a 75 130 bp fragment) to eliminate all the functional domains of the YAP1 protein (Figure 6A). PCR analyses showed that ≈58% (14/24) of the picked clones for exon 1 targeting, ≈38% (8/21) of those for exon 4 targeting, and ≈50% (1/2) of those for exon 1–4 targeting had homozygous deletion (Figure S8A, Supporting Information). Deletion of exon 1 and exon 1–4 completely ablated the expression of YAP1 protein, while those targeting exon 4 only resulted in formation of a mutated protein of smaller size (Figure 6B). The exon 4 targeting cells showed no reduction in *YAP1* mRNA expression and did not affect *GATA3* expression during differentiation (Figure S8B, Supporting Information). Therefore, the *YAP1* knockout cells with deletion of exon 1 or exon 1–4 were mainly used in subsequent experiments.

*YAP1* knockout did not affect the proliferation rates of undifferentiated hEPSC‐em though the growth rates of the *YAP1* knockout hEPSC‐em were lower than that of the wildtype cells during early trophoblast differentiation (Figure S8C, Supporting Information). Besides, *YAP1* knockout had no effect on pluripotency of hEPSC‐em, as demonstrated by unaltered expression of the pluripotent marker OCT4 and expression of three germ layer markers (muscle actin, *β*3 tubulin, and alpha fetoprotein) in the EB formed (Figure S8D, Supporting Information). We failed to derive hTSC line from the *YAP1* knockout hEPSC‐em in TSC medium and observed massive death of non‐epithelial like cells in the derivation; apoptotic assay demonstrated a significantly higher proportion of apoptotic cells in the *YAP1* knockout cells than the wildtype cells during the first passage of hTSC conversion (Figure S8E, Supporting Information). In contrast, the wildtype cells produced epithelial cell‐like cells in the first passage (Figure 6C). Substantial expression of TSC markers (*GATA3*, *KRT7*, and *TFAP2C*) was induced in the wildtype cells in the first cell passage but barely detected in the *YAP1* knockout cells (Figure 6D).

Next, we induced trophoblast differentiation using the BAP protocol. We noticed an induction of cytoplasmic phosphor‐YAP1 expression in the wildtype hEPSC‐em at 72 h, which was concordant with the decrease of nuclear YAP1 expression levels at this time point (Figure S8F, Supporting Information). The *YAP1* null cells expressed significantly less *GATA3*, *KRT7* (Figure 6E), *GATA2*, and *TFAP2C* (Figure S9A, Supporting Information) than the wildtype cells. Their expression of STB (*ERVW‐1*) and EVT (*HLA‐G* and *MMP2*) markers were also significantly reduced during differentiation (Figure S9A, Supporting Information). The *YAP1* knockout did not affect the expression of *TEAD1* and *TEAD3* (Figure S9A, Supporting Information), but significantly downregulated that of *AMOT* (Figure 6E) during differentiation. Flow cytometry showed a significant reduction of the GATA3^+^ cells at 72 h and the KRT7^+^ cells at 48 and 72 h post‐BAP treatment in the *YAP1* knockout cells when compared to the wildtype cells (Figure S9B, Supporting Information), demonstrating reduced trophoblast differentiation after deletion of *YAP1*.

The *YAP1* null hEPSC‐em could not form BAP‐EB and most of the spheroids formed were disaggregated from 24 h post‐treatment onwards with some remained as cell clumps (Figure S9A, Supporting Information), which expressed weak cytoplasmic but not nuclear GATA3 signal, in contrast to the nuclear GATA3 signal in outer layer of cells in the 48 h BAP‐EB from wildtype cells (Figure 6F). Interestingly, aPKC, the upstream regulator of *YAP1*,^[^ ^33^ ^]^ was expressed only on the apical side of the outer cells of 48 h BAP‐EB from the wild‐type cells. In the *YAP1* knockout cell clumps, aPKC signal was observed in both inner and outer cells of the clumps (Figure 6F), suggesting that cell polarity might have been affected. In addition, the AME marker, VIM^[^ ^20^ ^]^ was not expressed in the BAP‐EB derived from the wildtype and knockout cells (Figure S9B, Supporting Information). As the *YAP1* null hEPSC‐em could not form BAP‐EB, we conducted trophoblast spheroid attachment assay using BAP‐EB from hEPSC‐em with mutated exon 4 of *YAP1* only. The cells formed trophoblast spheroids but with significantly lower cyst formation rate and attachment rate on the receptive Ishikawa cells (Figure S9C, Supporting Information) when compared to those from the wildtype cells.

## 3 Discussion

In this study, we established EPSC directly from human pre‐implantation embryos using culture conditions adopted from our previous report.^[^ ^5^ ^]^ The hEPSC‐em were derived from either morulae or early blastocysts. The efficiency of hEPSC‐em derivation from human embryos was around 6%, which was similar to previous studies on derivation of primed hESCs.^[^ ^34^, ^35^ ^]^ The derivation rate is low when compare with derivation of EPSC from fresh mouse and porcine embryos.^[^ ^5^ ^]^ The derivation medium used in the present study was based on the derivation protocol of porcine embryos, which may not be optimal for human embryos. However, the limited availability of human embryos for research forbids extensive optimization of the derivation protocol. The suboptimal derivation condition may account for the low derivation rate in the present study. Attempts to create hEPSCs from cleavage stage embryos failed, suggesting that their creation may require modulation of other signaling pathways.

The established hEPSC‐em showed unique H3K4me3 broad domain conformation involving Hippo signaling molecules when compared with hEPSC‐ES, naive hESC and primed hESC. The observed H3K4me3 conformation in the hEPSC‐em was associated with enhanced trophoblast differentiation potency. Similar to hESC, the hEPSC‐em could be differentiated into embryonic lineages including PGCLC using conditions established in hESC.^[^ ^6^, ^8^ ^]^ It is noted that hEPSC‐em and hEPSC‐ES exhibited distinct H3K4me3 patterns though they were cultured in the same medium. During hEPSC‐em derivation, activin A and bFGF were included in the medium for better outgrowth of the human blastomeres. Both components were removed from the medium when colonies were seen (<4 days). The EPSC medium does not contain activin A and bFGF. On the other hand, hESC/hiPSC were cultured in medium containing bFGF. Early study demonstrated that the hESC adapted to the culturing condition in long term culture and change of culturing system could induce irreversible changes of part of the transcriptomes and DNA methylomes.^[^ ^36^ ^]^ Therefore, we speculated that at least part of the observed difference between the hEPSC‐em and the hEPSC‐ES in EPSC medium could be due to incomplete conversion of epigenetic marks in the hESC/hiPSC imposed by long term culture in bFGF containing medium. Moreover, as the hEPSC‐em lines were derived from individual human embryos, the possibility that differences in genetic/epigenetic background between the parental cells causing the observed differences in differentiation potency could not be excluded. Unfortunately, the possibility could not be addressed now as the number of cells in individual cleavage stage pre‐implantation embryos was too few to split for derivation of both the EPSC and the hESC from the same embryo in view of the low derivation efficiency.

PCA analysis of the transcriptomes revealed close clustering of hEPSC (hEPSC‐em and hEPSC‐ES) with human embryo at E4 stage but distinct from the primed hESCs. The current study and our published^[^ ^5^ ^]^ and unpublished data indicate that human EPSC have some of the transcriptomic features of the human embryos at morula stage. Nevertheless, we acknowledge that the EPSCs are in vitro cultured stem cells and are substantially different from the in vivo embryo cells in both molecular properties and developmental potential.

Although the global transcriptomes were similar between hEPSC‐em and naive hESC, there were differences in expression of specific cluster of genes between the two cell types. For instance, the expression of histone cluster genes and genes related to DNA methylation and Wnt pathway were higher while that of stem cell differentiation related genes were lower in the hEPSC‐em than the naive hESC. The two cell types also exhibited difference in histone methylation pattern, especially the H3K4me3 broad peaks. It is noted that EPSCs of human, pig, and bovine have high core histone protein levels and high DNA repair capacity.^[^ ^5^, ^37^ ^]^ in particular homologous recombination activities, which are reflected in efficient gene targeting of hEPSC‐em. Consistently, we identified a significant upregulation of DNA damage repair genes *GNL1* and *TLK2* in the hEPSC (hEPSC‐em and hEPSC‐ES) when compared to the naive hESC and the primed hESC. *GNL1* (Guanine nucleotide binding protein like 1) promotes cancer cell proliferation by modulating G1/S and G2/M phase transition,^[^ ^38^, ^39^ ^]^ while *TLK2* (Tousled‐like kinases 2) is important in chromatin assembly and maintenance of replication fork integrity. Knock‐out of *TLK2* resulted in loss of DNA replication and cell cycle arrest in the G1 phase.^[^ ^40^ ^]^ Our unpublished data also show normal genomic imprinting in human EPSCs.

The major difference in culturing naive hESC and hEPSC is that human naive cell culture conditions always contain the MEK1/2 inhibitor PD0325901 whereas our current human EPSC media have removed it. PD0325901 and inhibiting MEK1/2 cause abnormal genomic imprinting in human stem cells.^[^ ^41^, ^42^ ^]^ Our very first EPSC culture condition was developed based on the widely used 2i/LIF medium for culturing mouse naive ESC, which contains PD0325901.^[^ ^43^ ^]^ This condition was suitable for both mouse and human EPSC culture.^[^ ^44^ ^]^ However, we later found that PD0325901 was not suitable for culturing porcine and bovine pluripotent stem cells, and thus removed PD0325901 from the culture medium.^[^ ^5^, ^37^ ^]^ Critically, removal of PD0325901 does not seem to impair the potential of hEPSC to generate bona fide TSCs in our previously published data and a recent paper.^[^ ^5^, ^45^ ^]^

Human naive stem cell culture conditions are based on the 2i/LIF culture medium but have evolved in the past several years. The first two widely tested naive conditions are developed by Prof. Austin Smith^[^ ^46^ ^]^ and Prof. Rudolf Jaenisch.^[^ ^18^ ^]^ The Smith medium is updated to PXGL which additionally contains XAV939.^[^ ^47^ ^]^ Adding XAV939 in the naive cell medium eliminates a subpopulation of cells expressing high GATA2 in the original naive cell cultures.^[^ ^48^, ^49^ ^]^ Another naive cell condition was recently reported by Prof. Jacob Hanna,^[^ ^50^ ^]^ which also contains XAV939 or IWR‐1 and an inhibitor for SRC. Although these new naive stem cell conditions blur the line with EPSCs, they all contain PD0325901. Furthermore, none of the current human naive cell conditions is capable of establishing pluripotent stem cell lines from pre‐implantation embryos of large animals such as pig and bovine.

The histone methylation H3K4me3 patterns in relation to pluripotency control of the hEPSC‐em are similar to that in the hEPSC‐ES,^[^ ^5^ ^]^ the naive hESC, and the primed hESCs.^[^ ^51^ ^]^ For instance, the promoters of pluripotent markers (*OCT4*, *NANOG*, and *SOX2*) were marked by the permissive H3K4me3. In depth analysis revealed that the H3K4me3 marked important pathways in all the stem cell types studied, including the MAPK and the Wnt signaling. The former is essential for maintenance of hESC and their inhibition leads to loss of pluripotency,^[^ ^52^ ^]^ while the latter is essential for maintaining pluripotency in naive hESC.^[^ ^53^ ^]^ Enrichment of the Wnt signaling pathway in the analyses is understandable as the GSK3*β* inhibitor, CHIR99021 is included in the hEPSC and the naive hESC culture media.

Genes bound with the H3K4me3 marks in the broad domains are highly associated with chromatin accessibility and open chromatin conformation.^[^ ^54^ ^]^ They are poised for gene activation upon appropriate stimulation. The open chromatin structure in hEPSC‐em is associated with enhanced trophoblast differentiation potency in terms of enhanced trophoblast gene expression during induced trophoblast differentiation and success in derivation of hTSC from hEPSC‐em. On the other hand, the primed hESC with less H3K4me3 bound broad domains could not support hTSC derivation.^[^ ^55^ ^]^ The hTSC derived from hEPSC‐em are bona‐fide trophoblastic cells with the needed characteristics,^[^ ^56^ ^]^ including expression of trophoblast markers (*GATA3* and *KRT7*), C19MC miRNAs, and lack of HLA‐A, ‐B, and ‐C expression. The hTSC‐em form trophospheres in TSC medium. Unlike the BAP‐EB spheroids from hEPSC‐em, the trophospheres could not attach onto the receptive endometrial epithelial cells, indicating that they were not at a TE stage but likely similar to the post‐implantation CTB. The result is in line with the lack of endometrial cell attachment potential of blastocyst‐derived TSC.^[^ ^27^, ^28^ ^]^ To this end, we adapted the BAP protocol to capture the initial TE induction from hEPSC.

In the present study, BAP induced expression of the TE/trophoblast genes of pre‐^[^ ^13^ ^]^ and post‐implantation^[^ ^21^ ^]^ embryos from d4 post‐treatment, the STB and EVT genes from d6 onwards. It is known that the AME and the TE/trophoblast express a number of common genes including HCG and HLA‐G.^[^ ^14^, ^20^ ^]^ Two recent reports showed that the primed hESC and hEPSC cells differentiate into AME‐like cells upon exposure to BMP.^[^ ^14^, ^20^ ^]^ In human embryos, the EPI differentiates into AME on E10.^[^ ^57^ ^]^ Unexpectedly, we found high expression of the human post‐implantation AME specific gene set on EPI cell type in E7 pre‐implantation human embryos. The observations are in line with the suggestion that the exact gene signature of the human AME cells remains to be clarified.^[^ ^23^ ^]^ Indeed, it was reported that the current reported post‐implantation human AME cells^[^ ^21^ ^]^ were transcriptionally more similar to the TE cells rather than the AME cells of monkeys.^[^ ^23^ ^]^ We therefore also compared the transcriptome of BAP‐treated hEPSC‐ES with the cynomolgus AME signature^[^ ^24^ ^]^ and found that a subset of genes like *NTRK2* and *KCNMA1* were only induced at the late stage of BAP‐induced differentiation (data not shown).

Brachyury (*T*) is a transcription factor highly upregulated during formation of AME.^[^ ^58^ ^]^ Our BAP protocol did not induce the expression of Brachyury (*T*) (data not shown) but induced that of trophoblast specific genes. For instance, *ELF5* promoter de‐methylation and upregulation of ELF5 expression are features of placental hTSC.^[^ ^9^, ^59^ ^]^ In contrast, ELF5 is not expressed in the 9‐week human fetal and post‐implantation AME cells.^[^ ^21^, ^60^ ^]^ In this study, we observed higher expression of ELF5 in the hEPSC than the primed hESC upon BAP treatment. It is likely that the BAP protocol induced differentiation of hEPSCs mainly into the TE/trophoblast during early differentiation. Although trophoblast induction of the em lines were all higher than the prime ESCs, variations of the induction efficiencies were observed among the four hEPSC‐em lines. Sex‐biased expression of both XY‐linked genes have been reported.^[^ ^13^ ^]^ Interestingly, a study showed sex differences in trophoblast differentiation from hESCs, where female cells exhibited higher numbers of upregulated genes and pathways critical for trophoblast cell development.^[^ ^61^ ^]^ Coincidentally, higher trophoblast differentiation potency was observed in the female hEPSC‐em lines (hEPSC‐em3 and ‐em5) as compared to the male lines (hEPSC‐em1 and ‐em4).

Some Hippo signaling molecules are specifically bound by the H3K4me3 broad domains in the hEPSC‐em when compared to the other stem cells studied. Hippo signaling is crucial for the first lineage segregation in mouse embryos. In the outer cells of mouse morula, *Yap1* is translocated into the nucleus and forms a protein complex with *Tead4*, which activates *Cdx2* expression for TE formation.^[^ ^62^, ^63^ ^]^ The BAP‐EB model demonstrates upregulation of the Hippo signaling during trophoblast differentiation from hESC.^[^ ^28^ ^]^ In humans, *GATA3* and *YAP1* are colocalized to the outer cells of morula.^[^ ^64^ ^]^ A recent finding suggested critical roles of *TEAD4* and *CDX2* in trophoblast differentiation from hESCs.^[^ ^65^ ^]^ Consistently, Yap1 inhibitor impairs early trophoblast development in BAP‐EB.^[^ ^26^ ^]^ These observations prompt us to investigate the Hippo‐related molecules during trophoblast differentiation of hEPSC‐em.

Among the Hippo signaling molecules bound by the H3K4me3 broad domain in hEPSC‐em, almost all of them (*TEAD1*, *TEAD3*, *YAP1*, *WWC1*, and *YWHAE*) had high expression in the human TE. We hypothesized that the open chromatin conformation of Hippo signaling gene promoters in the hEPSC‐em contributes to their efficient transcription activation during TE/trophoblast differentiation. Here, we demonstrated enhanced expression of *TEAD1*, *TEAD3*, *YAP1*, and *WWC1* in the hEPSC‐em relative to the hEPSC‐ES during trophoblast differentiation. A recent study combining ATAC‐sequencing and RNA‐sequencing suggested that *TEAD1* and *TEAD3* are among the master regulators of TE specification in human embryos.^[^ ^2^ ^]^ Consistently, the cyst formation rate and the attachment competency of hEPSC‐em derived BAP‐EB were significantly higher than that from the hEPSC‐ES.

The role of Hippo signaling in human TE development and embryo attachment was largely unknown. We therefore studied the role of *YAP1* in trophoblast development by a loss‐of‐function approach. The prominent effects of exon 1 and exon 1–4 deletion on trophoblast differentiation demonstrated the crucial role of the TEAD‐binding domain. Intriguingly, trophoblast spheroid formation was completely abolished in hEPSC lacking the domain. The low cyst formation of BAP‐EB derived from hEPSC‐em with an intact exon 1 and a deleted exon 4 of *YAP1* supported the importance of the TEAD‐binding domain in TE development. Concordantly, a recent report using a gain‐of‐function approach showed that the TEAD‐binding domain of *YAP1* is essential for cyst formation of human blastoids.^[^ ^27^ ^]^ Here, we further demonstrated that the mutated *YAP1* could functionally reduce the attachment potential of BAP‐EB onto endometrial cells likely due to a defective TE differentiation. In addition, our results demonstrated that *YAP1* knockout led to failure of hTSC formation by leading to cell apoptosis. Since the pluripotency characteristics of *YAP1* knockout hEPSC‐em remained normal, we reasoned the effects could be trophoblast specific. Further studies are required to confirm this finding.

Our data support a role of YAP1 on trophoblast differentiation via *GATA3*. At 48 h post‐BAP induction, the mRNA expression of *GATA3* in the *YAP1* null cells was comparable to the wildtype cells, but the GATA3 protein expression in the former was weaker than the latter. Further spatial examination showed nuclear GATA3 signal in the wildtype BAP‐EB, but weak cytoplasmic GATA3 signal in the periphery cells of the YAP1 null BAP‐EB. In immune cells, cytoplasmic GATA3 indicate phosphorylation and ubiquitination of GATA3 that lead to degradation of the molecule.^[^ ^66^, ^67^ ^]^ Thus, the discrepancies between the *GATA3* mRNA and protein levels during BAP‐induced trophoblast differentiation in the *YAP1* null cells could be due to degradation of *GATA3* in the absence of *YAP1*. The post‐translational regulatory role of YAP1 on GATA3 in the context of trophoblast differentiation warrants further studies. At 72 h post‐induction, we observed further increase in the expression of *YAP1* mRNA, but decrease in the nuclear YAP1 protein signal. This was associated with an increase in cytoplasmic phospho‐YAP1. It is known that phospho‐YAP1 exhibits non‐transcriptional function in cell cycle progression.^[^ ^68^ ^]^ The function of phospho‐YAP1 in trophoblast differentiation remains to be explored.

AMOT and aPKC may mediate the suppressive effects of *YAP1* knockout on trophoblast differentiation and spheroid formation; downregulation of *AMOT* and relocation of the aPKC immunoreactivities in the residue cell clumps were observed during BAP‐EB formation of the *YAP1* knockout cells. Although *AMOT* is an upstream co‐activator of *YAP1*,^[^ ^69^ ^]^ YAP1 binds to the enhancer of *AMOT* and induces its expression in human cancer cells,^[^ ^70^ ^]^ suggesting a possible feedback control. In human and mouse morulae, the apical localization of AMOT and aPKC in the outer cells is responsible for inactivation of Hippo signaling and nuclear translocation of YAP1, which then together with TEAD4 promote the initiation of TE specification and cavitation of blastocysts.^[^ ^64^, ^71^ ^]^ Inhibition of aPKC activity suppresses TE formation in mouse^[^ ^72^ ^]^ and human^[^ ^64^ ^]^ embryos. Interestingly, our data suggested that *YAP1* knockout disrupted cell polarity as indicated by relocation of the aPKC immunoreactivities to the cell–cell interface of all cells in the cell clumps. It is not known how *YAP1* affects cell polarity. The observation warrants further research in understanding the mechanism of *YAP1* in affecting early TE development.

## 4 Conclusion

We established the hEPSC‐em directly from human pre‐implantation embryos. The hEPSC‐em had expanded potency competent of differentiating into embryonic and extra‐embryonic lineages. Compared with the primed ESC, naive hESC, and hEPSC‐ES, an open chromatin pattern with bound H3K4me3 was identified in the hEPSC‐em. We identified a set of Hippo signaling genes uniquely marked with the H3K4me3 broad domain in the hEPSC‐em and demonstrated that the Hippo signaling contributed to the enhanced trophoblast differentiation potency of hEPSC‐em.

## 5 Experimental Section

### hEPSC Culture

hEPSC were cultured and maintained as described^[^ ^5^ ^]^ with minor modifications. Briefly, the cells were cultured on mitomycin‐C (Thermo Fisher Scientific) inactivated STO feeder cells, which were seeded on 0.1% gelatin (Sigma Aldrich)‐coated wells at a density of 0.075 × 10^6^ cells/cm^2^ at least 2 days prior to hEPSC seeding. The hEPSC medium: DMEM/F12, 1× L‐glutamine, 1× penicillin–streptomycin, 1× NEAA, 0.1 µm 2‐mercaptoethanol, 1× N2 supplement, 1× B27 supplement (Thermo Fisher Scientific), and 65 µg mL^−1^ L‐ascorbic acid (Sigma Aldrich) supplemented with 2.5 µm XAV939 (Sigma Aldrich), 0.15 µm A419259 (Tocris); 1.0 µm CHIR99021 (Stemgent), 0.25 µm SB590885 (R&D), and 10 ng mL^−1^ recombinant human LIF (PeproTech). 20% Knock‐out serum replacement (KOSR; Thermo Fisher Scientific) and 10 µm Y‐27632 (Stemcell Technologies) were supplemented to the medium on the day of hEPSC seeding and hEPSC were passaged every 3–4 days.

### Derivation of hEPSCs from Donated Human Pre‐Implantation Embryos

Human pre‐implantation embryos were donated from patients who had completed their family after assisted reproduction treatment. Written informed consents were obtained from all the donors recruited and the study protocols were approved by the Institutional Review Boards of the University of Hong Kong/Hospital Authority Hong Kong West Cluster (IRB number: UW 18‐017) and the Council of Reproductive Technology, Hong Kong (research license number: R5004). Cryopreserved day 2 human pre‐implantation embryos were thawed and cultured in G‐1 medium (Vitrolife) for 1 day before transferred to G‐2 medium (Vitrolife) and cultured till morula or early blastocyst stages when the zona pellucida of the embryos was removed using acid tyrode solution (Sigma Aldrich). The human embryos were then placed on the STO feeder cells in hEPSC medium supplemented with 10 ng mL^−1^ recombinant bFGF (R&D), 20 ng mL^−1^ recombinant activin A (PeproTech), and 5% FBS (Thermo Fisher Scientific). The embryos showed outgrowth after several days. The resultant cell colonies were dissected into small clusters using glass pipettes and transferred onto new STO feeder cells. After several passages, the colonies were digested with 0.05% Trypsin (Thermo Fisher Scientific) and passaged like other hEPSCs.

### hESCs Culture

hESC lines VAL3 and H9 were obtained from the Spanish Stem Cell Bank and the WiCell Research Institute, respectively. They were maintained as previously described.^[^ ^25^ ^]^

### In Vivo Teratoma Formation

hEPSCs were injected subcutaneously into 6–8 weeks old NOD‐SCID mice. The teratomas were harvested 8–10 weeks later. They were fixed in 4% paraformaldehyde and then embedded in paraffin blocks. Paraffin‐embedded samples were cut at 5 µm thickness and mounted on slides. The animal experiments were performed in accordance with the Committee on the Use of Live Animals in Teaching and Research, The University of Hong Kong (CULATR, HKU; 4663‐18).

### Differentiation of hEPSC‐em into PGCLCs and Pancreatic Lineage

hEPSC‐em were differentiated into PGCLCs using previously established protocol^[^ ^6^ ^]^ with modifications. hEPSC‐em were induced to differentiate to pre‐ME for 12 h on fibronectin (16.7 µg mL^−1^)‐coated wells with a cell density of 0.05 × 10^6^ cells/cm^2^. The medium used contained Advanced RPMI 1640, 1% B27 supplement, 1× glutamine, 1× penicillin–streptomycin, 1× NEAA, 100 ng mL^−1^ recombinant activin A, 3 µm CHIR99021, and 10 µm Y‐27632. Finally, the pre‐ME cells were digested and seeded into ultra‐low attachment 96‐well plates (Corning) at a density of 4000 cells/well for PGCLC induction for 6 days. The PGCLC media contained Advanced RPMI 1640, 1% B27 supplement, 1× glutamine, 1× penicillin–streptomycin, 1× NEAA, 0.1 µm 2‐mercaptoethanol, 500 ng mL^−1^ recombinant BMP2, 10 ng mL^−1^ recombinant human LIF, 100 ng mL^−1^ recombinant mouse SCF, 50 ng mL^−1^ recombinant mouse EGF (PeproTech), 10 µm Y‐27632, and 0.25% poly‐vinyl alcohol (Sigma‐Aldrich).

hEPSC‐em were differentiated into DE and PP cells using the StemDiff Pancreatic Progenitor Kit as previously described.^[^ ^8^ ^]^

### BAP‐Induced Differentiation of hESCs and hEPSCs into Trophoblastic Lineage

hEPSCs and hESCs were differentiated into trophoblastic spheroids (BAP‐EB) as previously described^[^ ^25^ ^]^ with slight modifications. Briefly, the cells were digested and seeded in AggreWell 400 plates (Stemcell Technologies) at a density of 150 cells/EB for hEPSCs or 200 cells/EB for hESCs. After 24 h, the EBs were transferred to ultra‐low cell attachment 6‐well plates (Corning). The differentiation medium consisted of mouse embryonic fibroblast conditioned medium: MEF‐CM supplemented with 10 ng mL^−1^ BMP4 (R&D), 1 µm A83‐01 (Stemgent), and 0.1 µm PD173074 (Stemgent). In some experiments, the trophoblast differentiation was conducted in monolayer. The cells were digested and seeded onto Matrigel‐coated plates (Corning) at 0.025 × 10^6^ cells/cm^2^ in MEF‐CM. The differentiation medium was changed from the next day onwards. In some experiments, HES medium (knockout DMEM, 15% KOSR, 1× glutamine, 1× penicillin–streptomycin, 1× NEAA, and 0.1 µm 2‐mercaptoethanol) was used as the differentiation medium with supplementation of different doses of BAP: high (BMP4: 10 ng mL^−1^, A83‐01: 1 µm, PD173074: 0.1 µm), mid (BMP4: 0.5 ng mL^−1^, A83‐01: 0.5 µm, PD173074: 0.1 µm), and low (BMP4: 0.5 ng mL^−1^, A83‐01: 0.1 µm, PD173074: 0.1 µm).

### Derivation of TSC Lines from hEPSCs

hTSC lines were derived from hEPSCs according to a previous protocol^[^ ^9^ ^]^ with minor modifications. Briefly, hEPSCs were digested and seeded onto collagen IV (5 µg mL^−1^)‐coated wells. The hTSC medium consisted of DMEM/F12, 0.3% BSA, 0.2% FBS, 1% ITS‐X supplement, 1× glutamine, 0.5% penicillin–streptomycin, 0.1 µm 2‐mercaptoethanol and 50 µg mL^−1^ L‐ascorbic acid, 50 ng mL^−1^ recombinant EGF (R&D), 2 µm CHIR99021, 0.5 µm A83‐01, 1 µm SB431542 (Stemgent), 0.8 mm VPA (Wako), and 5 µm Y‐27632. After 3–4 days, the TSC‐like colonies appeared. They were then digested with TrypLE Express (Thermo Fisher Scientific) and passaged every 3–4 days. Stable TSC lines were formed after 8–10 passages.

The methods of differentiation of hTSC into STB and EVT reported previously^[^ ^9^ ^]^ were used with minor modifications. For the induction of STB, hTSC were seeded onto collagen IV (2.5 µg mL^−1^)‐coated wells at a density of 0.02 × 10^6^ cells/cm^2^. The STB medium consisted of DMEM/F12, 0.3% BSA, 4% KOSR, 1% ITS‐X supplement, 1× glutamine, 0.5% penicillin–streptomycin, 0.1 µm 2‐mercaptoethanol, 50 µg mL^−1^ L‐ascorbic acid, 2 µm forskolin (Wako), and 2.5 µm Y27632. For the induction of EVT, hTSC were seeded onto collagen IV (1 µg mL^−1^)‐coated wells at a density of 0.015 × 10^6^ cells/cm^2^. The EVT medium contained DMEM/F12, 0.3% BSA, 3 4% KOSR, 1% ITS‐X supplement, 1× glutamine, 0.5% penicillin–streptomycin, 0.1 µm 2‐mercaptoethanol, and 50 µg mL^−1^ L‐ascorbic acid supplemented with 100 ng mL^−1^ NRG1 (day 0–3; R&D Systems), 7.5 µm A83‐01, 2.5 µm Y27632, and Matrigel (2% for day 0–3, 0.5% for day 3–6). The expression of STB and EVT specific genes were analyzed up to day 6 post‐induction of differentiation.

### BAP‐EB Attachment Assay

The attachment of BAP‐EB formed from different conditions onto HEC1‐B and Ishikawa cells were performed as previously described.^[^ ^25^ ^]^

### Genome Editing of hEPSC Using CRISPR‐Cas9

For CRISPR/Cas9 mediated knock‐in, the single guide RNA target sequence (GATA3: 5′‐GCAAGTCGAAAGGGACTGCA‐3′) was inserted into the pSpCas9(BB)‐2A‐GFP vector (PX458; Addgene #48138). GATA3‐mCherry donor vector (3 µg) and Cas9‐gRNA vector (2 µg) were electroporated into hEPSC. For *YAP1* knock‐out, two pairs of gRNAs (exon 1: 5′‐GTGCACGATCTGATGCCCGG‐3′ and 5′‐GGGGCAACGAGGTTACCTGT‐3′; exon 4: 5′‐GATGAACCTTTACCAAAACG‐3′ and 5′‐AATTTCTCCATCCTGAGTCA‐3′) were inserted individually into the pKLV2‐U6‐gRNA‐PGK‐puro vector (Addgene #67974). The Cas9 vector (pKLV2‐EF1a‐Cas9Bsd; Addgene #68343; 6 µg) and the gRNA vectors (3 µg) were electroporated into hEPSC using a Neon Transfection System (Thermo Fisher Scientific) with 1 pulse of 1400 V for 20 ms. The electroporated cells were either FACS‐sorted or selected with antibiotics (blasticidin: 10 µg mL^−1^; puromycin: 2 µg mL^−1^). For reporter knock‐in, the positive cells were further selected by puromycin. To validate the correct genome modifications, the targeted regions were PCR amplified for Sanger sequencing at the Centre for PanorOmic Sciences (CPOS), the University of Hong Kong. The PCR and sequencing primers are listed in the Table S1, Supporting Information.

### Pyrosequencing

Bisulfite conversion of genomic DNA was performed with the EpiTect Bisulfite Kit (Qiagen). The targeted regions were amplified by PCR. The purified PCR products were sequenced with the PSQ 96MA (Biotage, Qiagen). The pyrosequencing results were analyzed using the Pyro Q‐CpG software (Biotage). The pyrosequencing service was provided by CPOS. The PCR and sequencing primers are listed in the Table S1, Supporting Information.

### Quantitative Polymerase Chain Reaction

Total RNAs were extracted by the mirVana PARIS Kit (Thermo Fisher Scientific) and converted to cDNA by the TaqMan Reverse Transcription Kit (Takara). Real time quantitative PCR (qPCR) was performed in an Applied Biosystems 7500 Real‐Time PCR System (Thermo Fisher Scientific) using the TaqMan Gene Expression Assay. Quantifications were determined by the 2^−ΔΔ^ *^CT^* method. The mRNA levels were normalized with the endogenous 18S ribosomal RNA. The qPCR primers are listed in the Table S1, Supporting Information.

### Immunofluorescence Staining

Cells were fixed in 4% paraformaldehyde. After permeabilization with 0.1% Triton X‐100 (Sigma‐Aldrich), they were incubated with appropriate blocking solution followed by the primary antibodies at 4 °C overnight. The cells were then incubated with fluorescence‐conjugated secondary antibodies (Thermo Fisher Scientific). The nucleus was stained with Hoechst 33258 (Thermo Fisher Scientific). Images of the stained cells were captured using a confocal microscope (LSM 800, Carl Zeiss AG) at the CPOS. The antibodies used in this study are listed in Table S2, Supporting Information.

### Fluorescence‐Activated Cell Sorting

Cells were fixed in 4% paraformaldehyde and permeabilized in 100% methanol. The cells were then incubated with primary antibodies for 1 h at room temperature, followed by incubation with fluorescence‐conjugated secondary antibodies for 1 h at room temperature. The cells were finally resuspended in PBS supplemented with 0.1% BSA and analyzed using BD LSR Fortessa Analyzer at the CPOS. The antibodies used in this study are listed in Table S2, Supporting Information.

### Cell Proliferation Assay

The cell proliferation assay was conducted with the XTT Cell Proliferation Kit (Sigma‐Aldrich). Cells grown in hEPSC or trophoblast differentiation medium were assayed for cell proliferation on each day. XTT labeling reagent was mixed with electron coupling reagent in a ratio of 50:1. The resulting mixture (100 µL) was added to the cells grown in 96‐well. The cells were incubated at 37 °C for 4 h, followed by determination of absorbance at 450 nm.

### Bioinformatics Analyses

hEPSC and BAP‐EB derived from hEPSC were subjected to scRNA‐seq by the Chromium Single Cell Gene Expression kit (10x Genomics). Single cell suspensions were encapsulated by the Chromium Single Cell 5′ Reagent Kit (10x Genomics). cDNA libraries were prepared by the Chromium Single Cell A Chip Kit (10x Genomics). An Illumina NovaSeq 6000 was used for Pair‐End 151 bp sequencing. The Cellranger mkfastq was used to demultiplex raw base call into FASTQ files. The Cellranger count was then used to process the FASTQ files for alignment, filtering, barcode counting, and UMI counting. The RNA‐seq data from published studies^[^ ^5^, ^12^, ^13^, ^14^, ^21^ ^]^) were obtained from GSE36552, GSE59435, E‐MTAB‐3929, and E‐MTAB‐6819. The gene list for stem cell differentiation was obtained from http://amigo.geneontology.org/amigo/term/GO:0048863). Differential gene expression analysis was performed with the R package linear model for microarray data (limma‐voom) with pre‐processing using trimmed mean of M values method.^[^ ^73^ ^]^ The batch effects of different datasets were normalized using ComBat.^[^ ^74^ ^]^ The principal components were computed and plotted with the R packages FactoMineR and factoextra.^[^ ^75^ ^]^ The heatmaps were plotted with gplots using *z*‐scores calculated for each gene across different samples. For the analysis of lineage specific genes from the human post‐implantation embryo dataset GSE136447^[^ ^21^ ^]^ upon re‐annotation,^[^ ^22^ ^]^ Seurat v4.0 was used. The single cells were further subset with nFeature_RNA > 500 and percent.mt < 10. The resulting dataset was normalized and scaled, while the lineage specific genes were identified by FindMarkers function. The common genes across lineages were removed prior to heatmap plotting.

hEPSC were also subjected to ChIP‐seq. Chromatin immunoprecipitation was first performed as previously described.^[^ ^8^ ^]^ The antibodies used for ChIP are listed in Table S2, Supporting Information. Total DNA input or 1 ng of antibody‐captured DNA was subjected to library preparation using the KAPA Hyper Prep Kit (Roche). An Illumina NovaSeq 6000 was used for Pair‐End 151 bp sequencing. The alignment was performed with BWA. The peak calling was performed with MACS2. The peak annotation was performed with HOMER. The ChIP‐seq data from published studies^[^ ^5^, ^18^ ^]^ were obtained from E‐MTAB‐7252 and GSE59435. Alignment bam files were converted into read coverage files in bigWig format for visualization using deepTools^[^ ^76^ ^]^ with the RPKM normalization method. The Integrative Genomics Viewer v2.11.1 was used for visualization of histone peaks. Gene ontology analysis was performed with Database for Annotation, Visualization and Integrated Discovery v6.8.^[^ ^77^ ^]^

### Statistical Analyses

Data were analyzed and plotted using the SigmaPlot software (Systat Software). Statistical analyses were performed using *t*‐test, rank sum test, or one way ANOVA where appropriate. A *p*‐value < 0.05 was considered as statistical significantly different.

### Ethics Approval statement

Written informed consents were obtained from all the donors recruited and the study protocols were approved by the Institutional Review Boards of the University of Hong Kong/Hospital Authority Hong Kong West Cluster (IRB number: UW 18‐017) and the Council of Reproductive Technology, Hong Kong (research license number: R5004). The animal experiments were performed in accordance with the Committee on the Use of Live Animals in Teaching and Research, The University of Hong Kong (CULATR, HKU).

## Conflict of Interest

The authors declare no conflict of interest.

## Supporting information

Supporting Information

## Data Availability

The data that support the findings of this study are available from the corresponding author upon reasonable request.
