# Supplementary material for: Expanded Potential Stem Cells from Human Embryos Have an Open Chromatin Configuration with Enhanced Trophoblast Differentiation Ability
Source: Adv Sci (Weinh). 2023 Feb 12;10(11):2204797. doi: 10.1002/advs.202204797 (PMC10104645; doi:10.1002/advs.202204797)
Supplement: Supplementary file 1 — Supporting Information [file ADVS-10-2204797-s001.pdf]

ADVANCED SCIENCE
Open Access

Supporting Information

for *Adv. Sci.*, DOI 10.1002/advs.202204797

Expanded Potential Stem Cells from Human Embryos Have an Open Chromatin Configuration with Enhanced Trophoblast Differentiation Ability

Andy Chun Hang Chen, Yin Lau Lee, Hanzhang Ruan, Wen Huang, Sze Wan Fong, Siyu Tian, Kai Chuen Lee, Genie Minju Wu, Yongqi Tan, Timothy Chun Hin Wong, Jian Wu, Weiyu Zhang, Dandan Cao, Judy Fung Cheung Chow, Pengtao Liu and William Shu Biu Yeung**

Supporting Information

Expanded potential stem cells from human embryos have an open chromatin configuration with enhanced trophoblast differentiation ability

Andy Chun Hang Chen[#,1,2,3], Yin Lau Lee[#,1,2,3], Hanzhang Ruan[1], Wen Huang[1], Sze Wan Fong[1], Siyu Tian[1], Kai Chuen Lee[1,3], Genie Minju Wu[1], Yongqi Tan[1], Timothy Chun Hin Wong[3], Jian Wu[3], Weiyu Zhang[3], Dandan Cao[2], Judy Fung Cheung Chow[1], Pengtao Liu[,3,4], William Shu Biu Yeung[*, 2,3,1]*

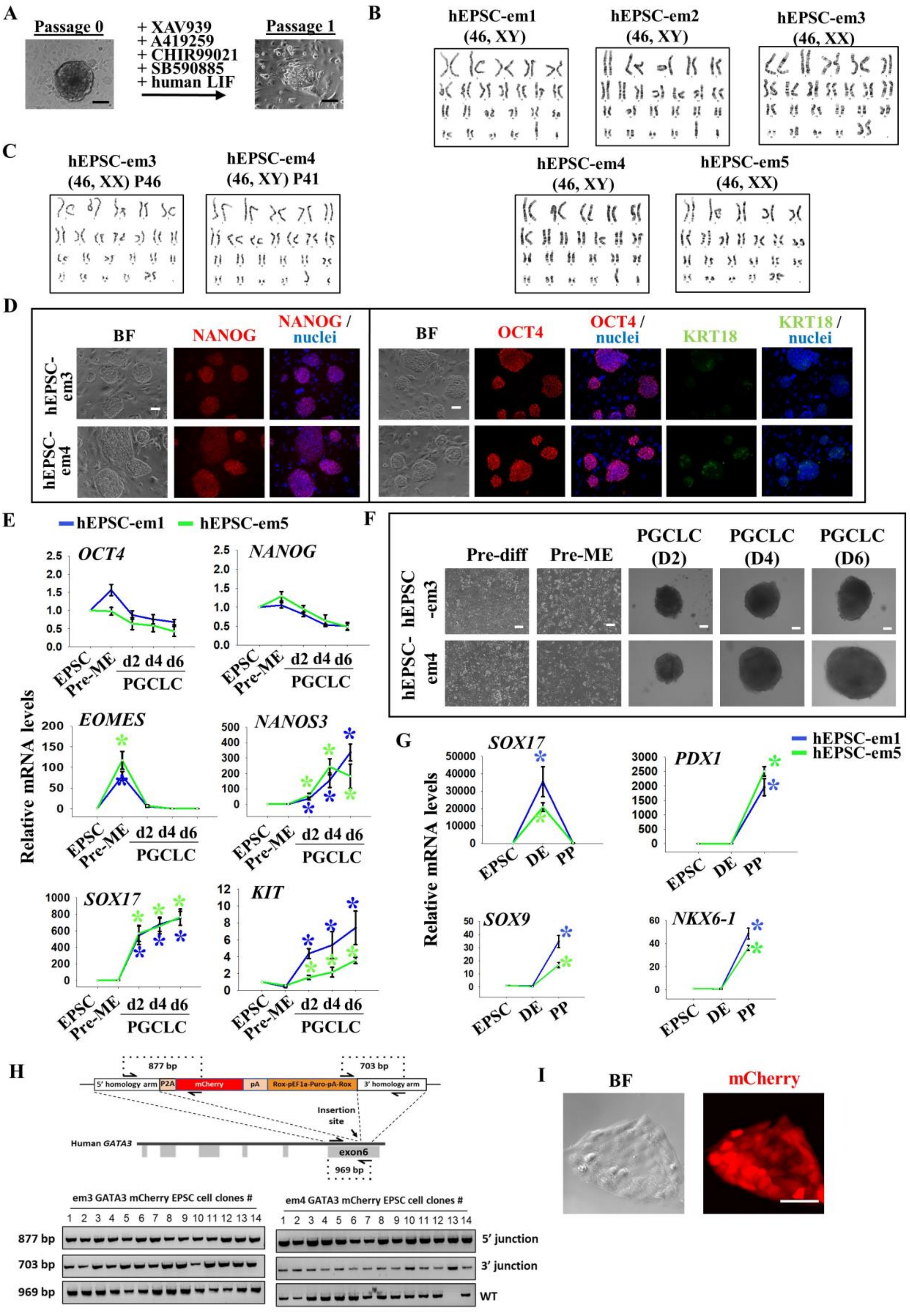

Fig. S1. Establishment of EPSC from human pre-implantation embryos (hEPSC-em)

(A): Images of donated human embryos at morula stage (left) and mechanically dissected colony (right) during the derivation of hEPSC-em. Scale bar: 100 μm. **(B):** Karyotyping of hEPSC-em-1, -2, -3, -4 and -5. **(C):** Karyotyping of hEPSC-em-3 and hEPSC-em-4 after long-term culture (>40 passages). **(D):** Immunofluorescent staining of OCT4, NANOG and KRT18 in undifferentiated hEPSC-em-3 and hEPSC-em-4. The nuclei were stained with Hoest33258. Scale bar: 100 μm. **(E):** RT-qPCR analysis of *OCT4*, *NANOG*, *EOMES*, *NANOS3*, *SOX17* and *KIT* during PGCLC formation from hEPSC-em1 and hEPSC-em5. *: $p<0.05$ compared to EPSC control, t-test; n=3. **(F):** Photos showing different stages of PGCLC differentiation from hEPSC-em. Pre-diff: pre-differentiation, Pre-ME: pre-mesendoderm. Scale bar: 100μm. **(G):** RT-qPCR analysis of DE marker (*SOX17*) and PP markers (*PDX1*, *SOX9* and *NKX6-1*) during pancreatic differentiation from hEPSC-em1 and hEPSC-em5. *: $p<0.05$ compared to EPSC control; t-test; n=3. **(H):** Schematic diagram showing knock-in of the mCherry reporter cassette after exon 6 of the *GATA3* locus (top). Arrow indicated the location of PCR primers for amplifying the 5’ and 3’ inserted regions, and the wildtype *GATA3* locus. PCR amplification of the 5’and 3’ inserted regions, and the wild-type GATA3 locus (bottom). **(I):** Images of mCherry reporter fluorescent signal in hTSC-em. Scale bar: 100μm.

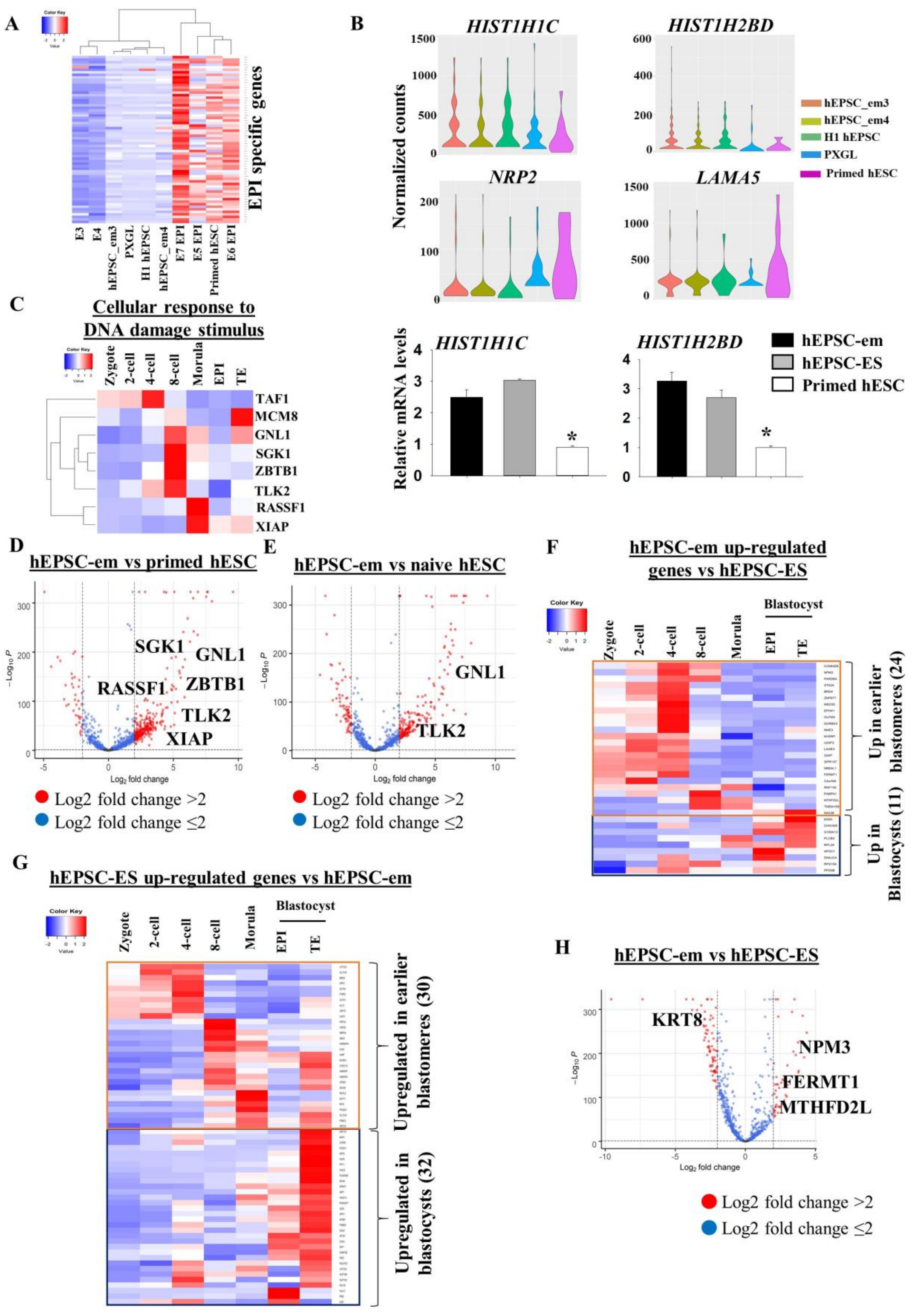

Fig. S2. Single cell RNA sequencing analysis of hEPSC-em transcriptomes

(A): Heatmap clustering showing the expression values of epiblast specific genes among hEPSC-em3, hEPSC-em4, hEPSC-ES, naive hESC, primed hESC, and human pre-implantation embryos. **(B):** The violin plots showing the expression patterns of histone cluster genes (*HIST1H1C*, *HIST1H2BD*) and stem cell differentiation genes (*NRP2*, *LAMA5*) between hEPSC-em, hEPSC-ES, primed hESC and naive hESC (top). RT-qPCR analysis of *HIST1H1C* and *HIST1H2BD* between hEPSC-em3, hEPSC-em4, hEPSC-ES and primed hESC. *: $p<0.05$ compared to EPSC control, t-test; n=4 (bottom). **(C)**: Heatmap showing the expression levels of genes under cellular response to DNA damage stimulus in human pre-implantation embryos. **(D)**: Volcano plot indicating the differentially expressed genes between hEPSC-em and primed hESC. *SGK1*, *GNL1*, *RASSF1*, *ZBTB1*, *TLK2* and *XIAP* were specifically labeled. **(E)**: Volcano plot indicating the differentially expressed genes between hEPSC-em and naive hESC. *GNL1* and *TLK2* were specifically labeled. **F-G**: Heatmap showing the expression levels of genes differentially upregulated in hEPSC-em **(F)** or in hEPSC-ES **(G)** in human pre-implantation embryos. **(H)**: Volcano plot indicating the differentially expressed genes between hEPSC-em and hEPSC-ES. *KRT8*, *NPM3*, *FERMT1* and *MTHFD2L* were specifically labeled. Published scRNA-seq data sets of human pre-implantation embryos (12), primed hESC (12), naive hESC (14) and hEPSC-ES (4) were used for the analysis.

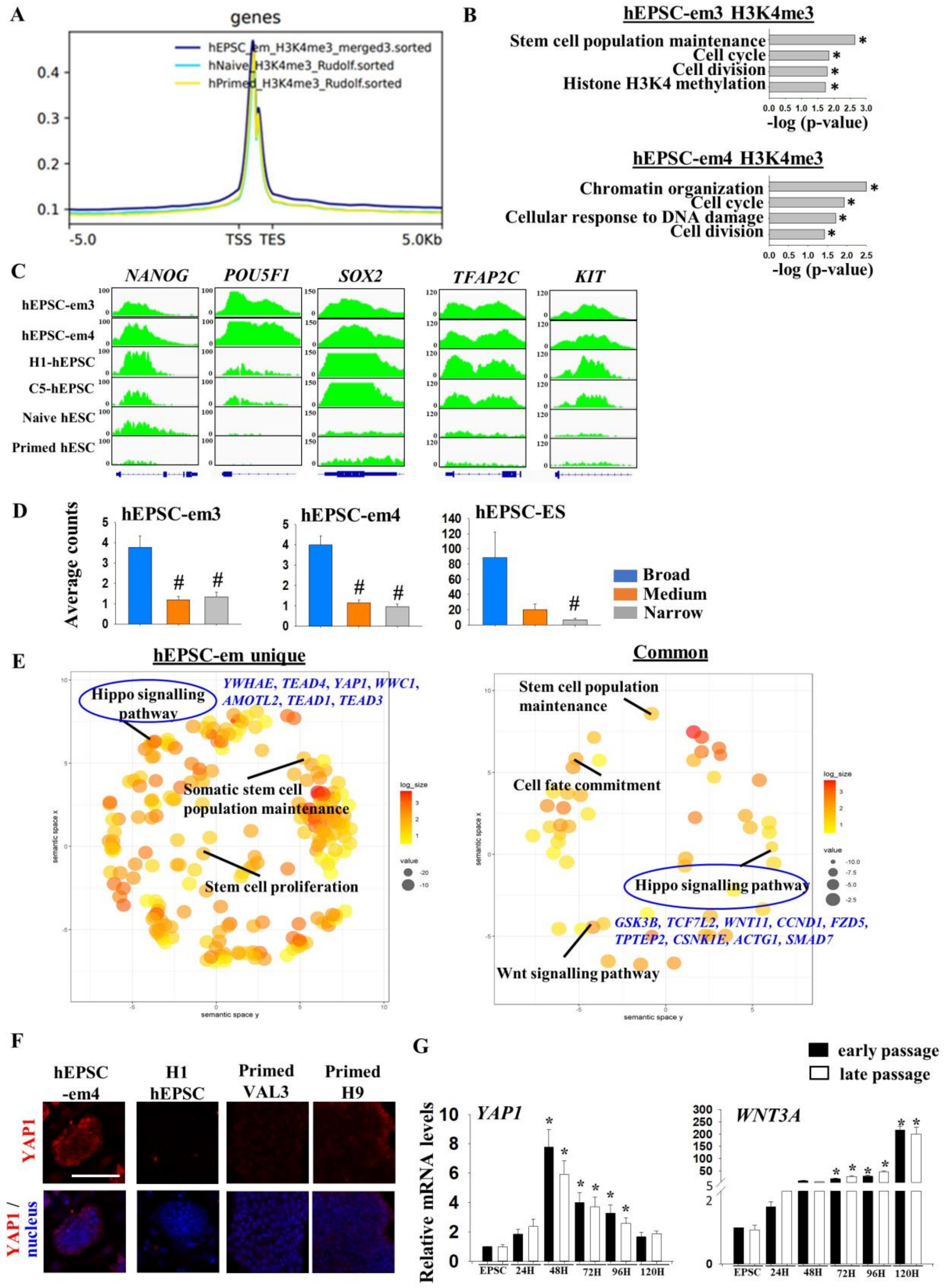

Fig. S3. hEPSC-em had unique broad H3K4me3 conformation

(A): Peak coverage plot from chromatin immunoprecipitation-sequencing (ChIP-seq) analysis of H3K4me3 marks in hEPSC-em3, hEPSC-em4, primed and naive hESC. The plot covered peaks around the transcription start site (TSS ± 5000bp). The ChIP-seq data of hEPSC-ES and

primed hESC were from Ref. 5 and 19 respectively. (**B**)**:** Gene ontology analysis for H3K4me3 marked gene promoters in hEPSC-em3 and hEPSC-em4. (**C**)**:** The tracks showing H3K4me3 peaks at the gene loci (*NANOG*, *POU5F1*, *SOX2*, *TFAP2C*, *KIT*) in hEPSC-em, hEPSC-ES, primed and naive hESC. (**D**)**:** Average counts of genes with broad (>5kb), medium (1-5kb) and narrow (<1kb) H3K4me3 domains between hEPSC-em and hEPSC-ES. #: $p<0.001$ compared to broad domain, t-test. scRNA-seq data of hEPSC-ES was from Ref. 5. (**E**)**:** REVIGO analysis of gene ontology enrichment in H3K4me3 broad domain unique in hEPSC-em (left) and common among different cell types (right). The genes of the Hippo signaling pathways were shown. **(F):** Immunofluorescent staining of YAP1 in undifferentiated hEPSC-em4, hEPSC-ES, naive and primed hESCs. The nuclei were stained with Hoest33258. Scale bar: 100 μm. **(G):** RT-qPCR analysis of *YAP1* and *WNT3A* upon trophoblast differentiation from hEPSC-em4 at early (20-40) and late (>50) passages. *: $p<0.05$ compared to EPSC control, t-test; n=3.

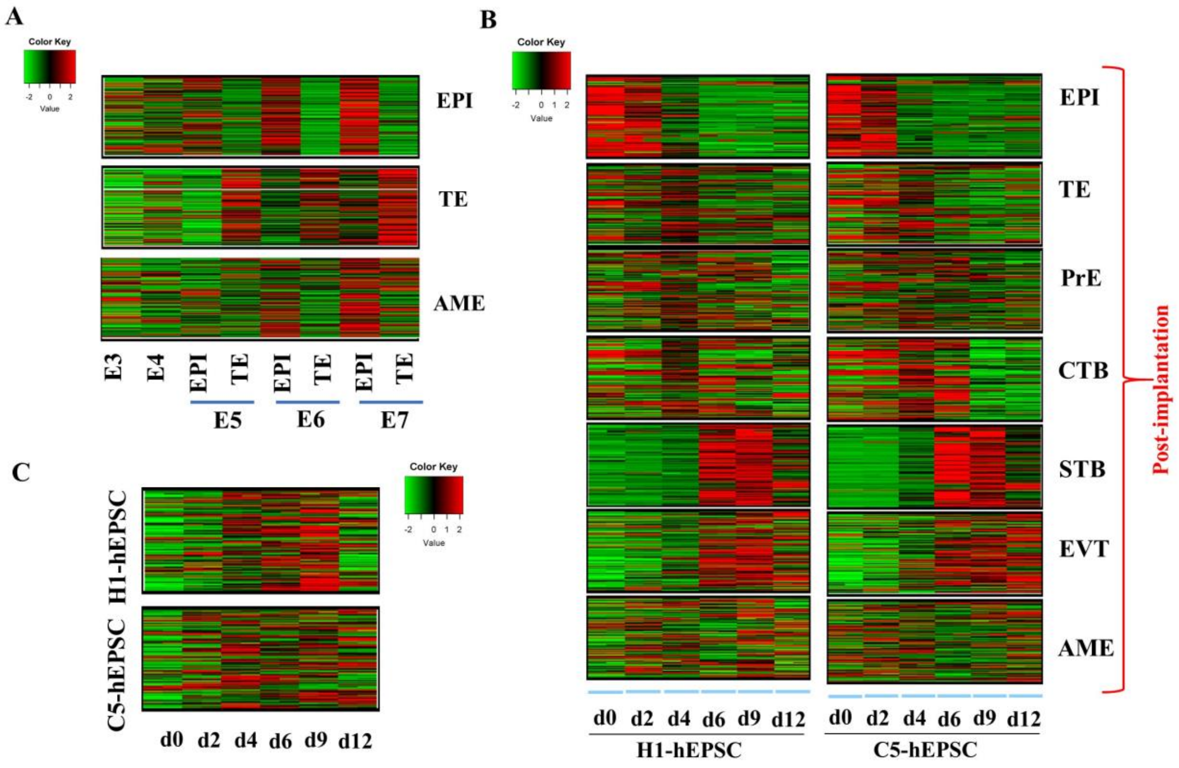

Fig. S4. Trophoblast differentiation from hEPSC-ES corresponded to trophoblast rather than amnion cells

(A): Heatmap showing the gene expression patterns in human pre-implantation embryos (Ref. 13) under the category of EPI, TE and AME in post-implantation embryos (Ref. 23). (**B**)**:** Heatmap showing the gene expression patterns during trophoblast differentiation from hEPSC-

ES (left: H1-hEPSC, right: C5-hEPSC) under the category of EPI, TE, Primitive endoderm (PrE), CTB, STB, EVT, AME in human post-implantation embryos from Ref. 23 after cell re-annotation as described in Ref. 24. (**C**): Heatmap showing the gene expression patterns during trophoblast differentiation from hEPSC-ES (top: H1-hEPSC, bottom: C5-hEPSC) under the category of cynomolgus AME from Ref. 26 after cell re-annotation as described in Ref. 24.

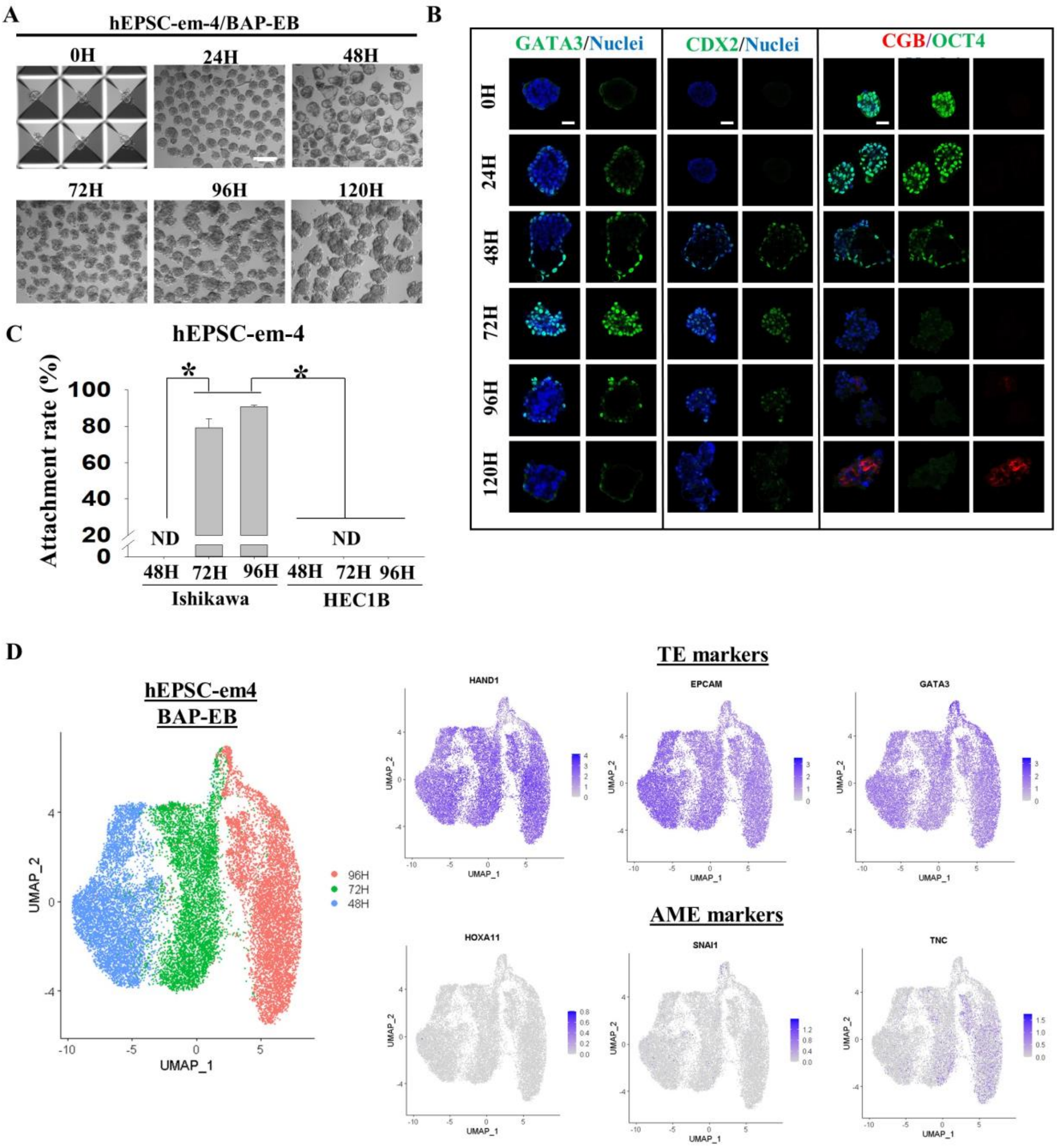

Fig. S5. BAP-EB formation from hEPSC-em

(A): Representative figures showing the trophoblast spheroids (BAP-EB) differentiated from hEPSC-em4 at different time points. Cystic structures were observed at 48H. Scale bar: 100μm. (**B):** Immunofluorescent staining of GATA3, CDX2, OCT4 and HCG of BAP-EB at different time points of BAP-EB differentiation from hEPSC-em4. The nuclei were stained with Hoest33258. Scale bar: 100μm. **(C)**: The attachment rate of hEPSC-em4 BAP-EB onto Ishikawa and HEC1B cells. *: $p<0.05$, t-test; n=4. **(D):** single-cell RNA sequencing analysis on TE markers (*HAND1*, *EPCAM* and *GATA3*) and AME markers (*HOXA11*, *SNAI1* and *TNC*) in BAP-EB derived from hEPSC-em4.

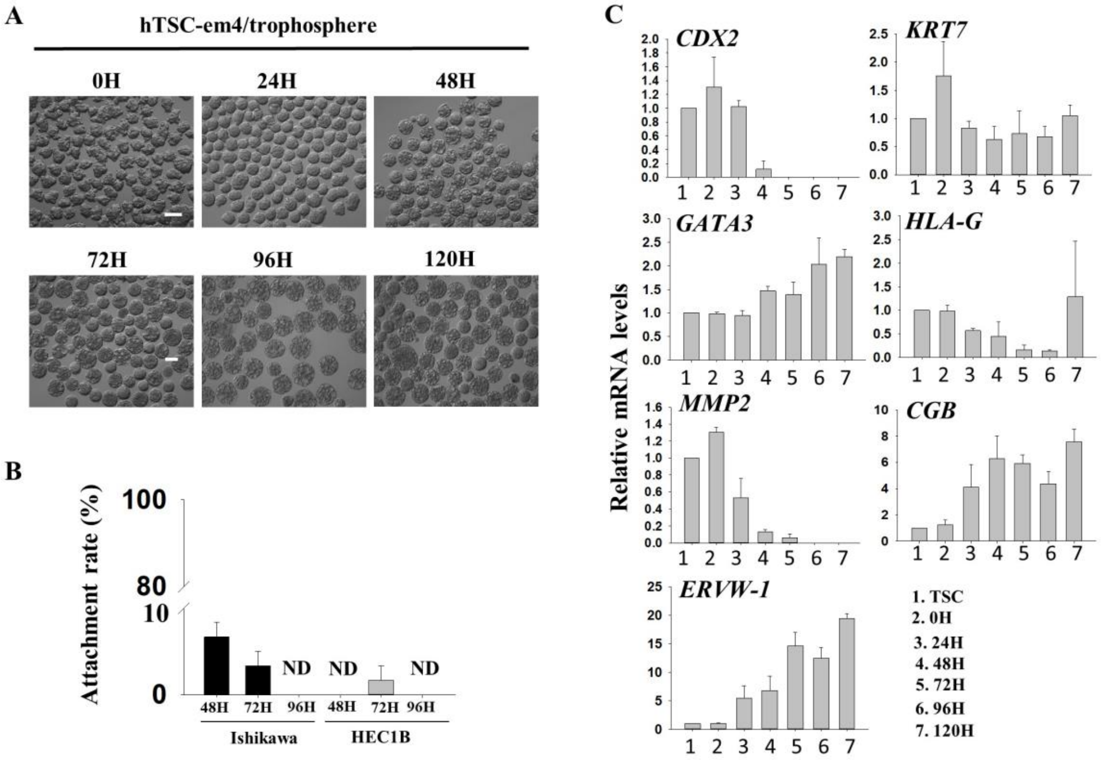

Fig. S6. Trophospheres formed from hTSC-em

(A): Representative figures showing the trophospheres formed from hTSC-em4 at different time points. Scale bar: 100μm. (**B):** The attachment rates of trophospheres -48h, -72h and -96h on receptive Ishikawa cells or non-receptive HEC-1B cells. n=4. ND: not detected. **(C):** RT-qPCR analysis of *CDX2*, *KRT7*, *GATA3*, *HLA-G*, *MMP2 CGB* and *ERVW-1* gene expressions in trophospheres formed from hTSC-em4; n=4.

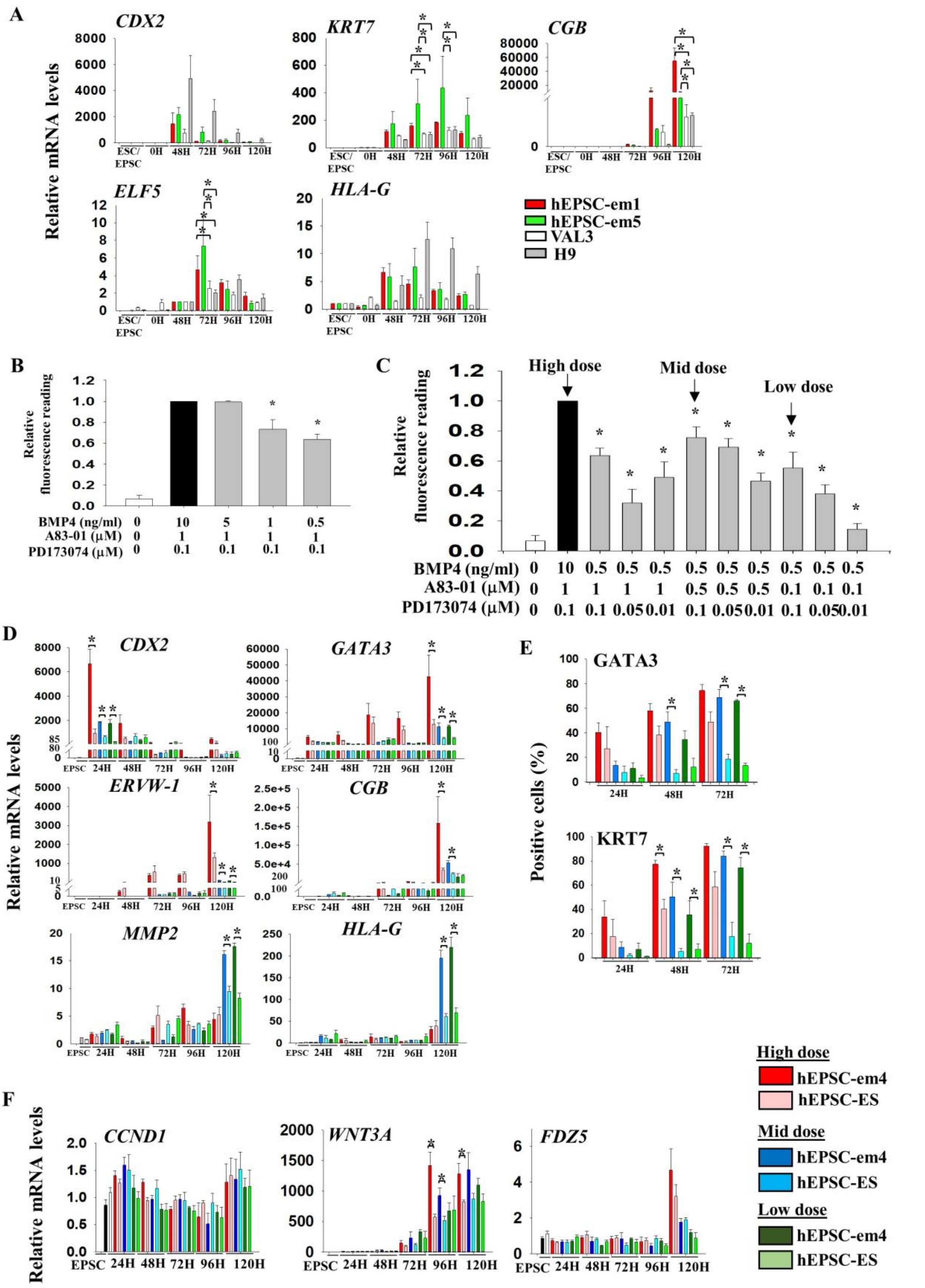

Fig. S7. hEPSC-em had enhanced trophoblast differentiation potency than hEPSC-ES

(**A**)**:** RT-qPCR analysis of *CDX2*, *KRT7*, *CGB*, *ELF5* and *HLA-G* gene expressions in BAP-EB formed from hEPSC-em1 and hEPSC-em5; n=3. (**B**)**:** Relative fluorescent reading of

GATA2-Venus reporter from hiPSC-EPSC upon trophoblast differentiation for 120H under different doses of BMP4 (0.5 ng/ml – 10 ng/ml) with constant A83-01 (1 μM) and PD173074 (0.1 μM). *: $p<0.05$ compared to high dose BAP (black bar); t-test; n=3. (**C**)**:** Relative fluorescent reading of *GATA2*-Venus reporter from hiPSC-EPSC upon trophoblast differentiation for 120H under different doses of BAP. *: $p<0.05$ compared to high dose BAP (black bar); t-test; n=3. **(D):** RT-qPCR analysis of *CDX2*, *GATA3*, *ERVW-1*, *CGB*, *MMP2* and *HLA-G* for trophoblast differentiation from hEPSC-em-4 and hEPSC-ES at different time points under different doses of BAP: high dose (BMP4: 10 ng/ml, A83-01: 1 μM, PD173074: 0.1 μM), mid dose (BMP4: 0.5ng/ml, A83-01: 0.5 μM, PD173074: 0.1 μM) and low dose (BMP4: 0.5 ng/ml, A83-01: 0.1 μM, PD173074: 0.1 μM). *: $p<0.05$ comparing between experimental groups; t-test; n=3. (**E**)**:** FACS analysis showing proportion of GATA3- and KRT7-positive cells after trophoblast differentiation from hEPSC-em-4 and hEPSC-ES at different time points under different doses of BAP. *: $p<0.05$ comparing between experimental groups; t-test; n=3. **(F):** RT-qPCR analysis of *CCND1*, *WNT3A* and *FDZ5* for trophoblast differentiation from hEPSC-em-4 and hEPSC-ES at different time points under different doses of BAP. *: $p<0.05$ comparing between experimental groups; t-test; n=3.

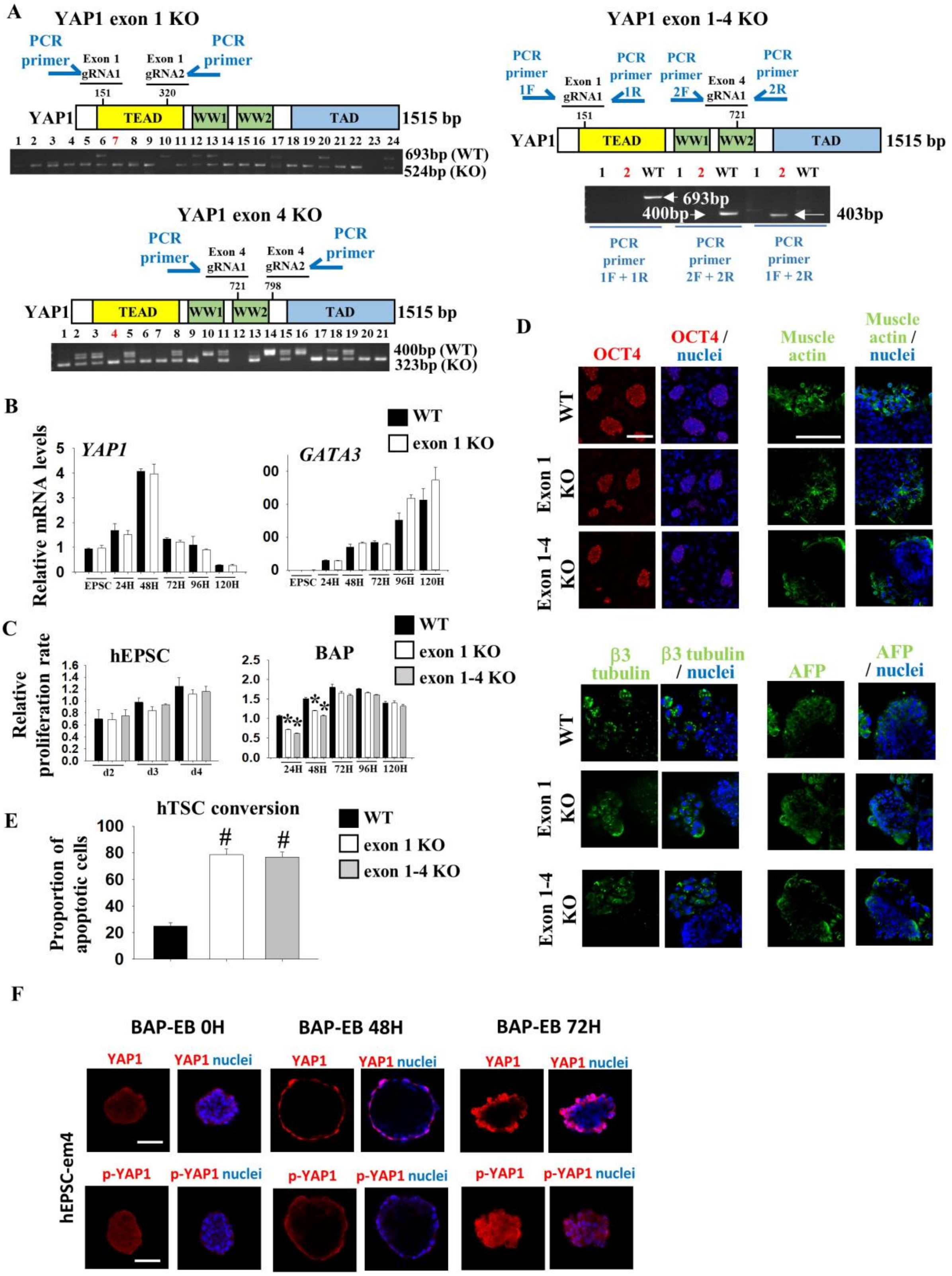

Fig. S8. YAP1 knockout inhibited trophoblast differentiation from hEPSC

(A): Schematic diagram showing the positions of gRNAs and PCR primers at exon 1, exon 4, and exon 1-4 of human *YAP1* gene. PCR analyses for validation of *YAP1* knockout were shown. The clones selected for subsequent studies were highlighted in red. (**B):** RT-qPCR analysis of

YAP1 and *GATA3* for trophoblast differentiation from hEPSC-em-4 wild-type and *YAP1* knockout cells at exon 4. n=3. (**C):** Proliferation rates of hEPSC-em-4 wild-type and *YAP1* knockout cells at exon 1 and from exon 1-4 in non-differentiated states (hEPSC) and during trophoblast differentiation (BAP differentiation) *: $p<0.05$ comparing with wild-type; t-test; n=3. (**D):** Immunofluorescent staining of OCT4 in hEPSC-em-4 wild-type and *YAP1* knockout hEPSC. Immunofluorescent staining of muscle actin (mesoderm); βIII tubulin (ectoderm); and alpha-fetoprotein (endoderm) in embryoid bodies derived from hEPSC-em-4 wild-type and *YAP1* knockout hEPSC-em. The nuclei were stained with Hoest33258. Scale bar: 100μm. (**E):** FACS analysis showing proportion of apoptotic cells after hTSC formation from hEPSC-em-4 wild-type and *YAP1* knockout cells at exon 1 and from exon 1-4. *: $p<0.05$ comparing with wild-type; t-test; n=4. **(F):** Immunofluorescent staining of YAP1 and phospho-YAP1 in BAP-EB from hEPSC-em-4 wild-type and *YAP1* knockout hEPSC. The nuclei were stained with Hoest33258. Scale bar: 100μm.

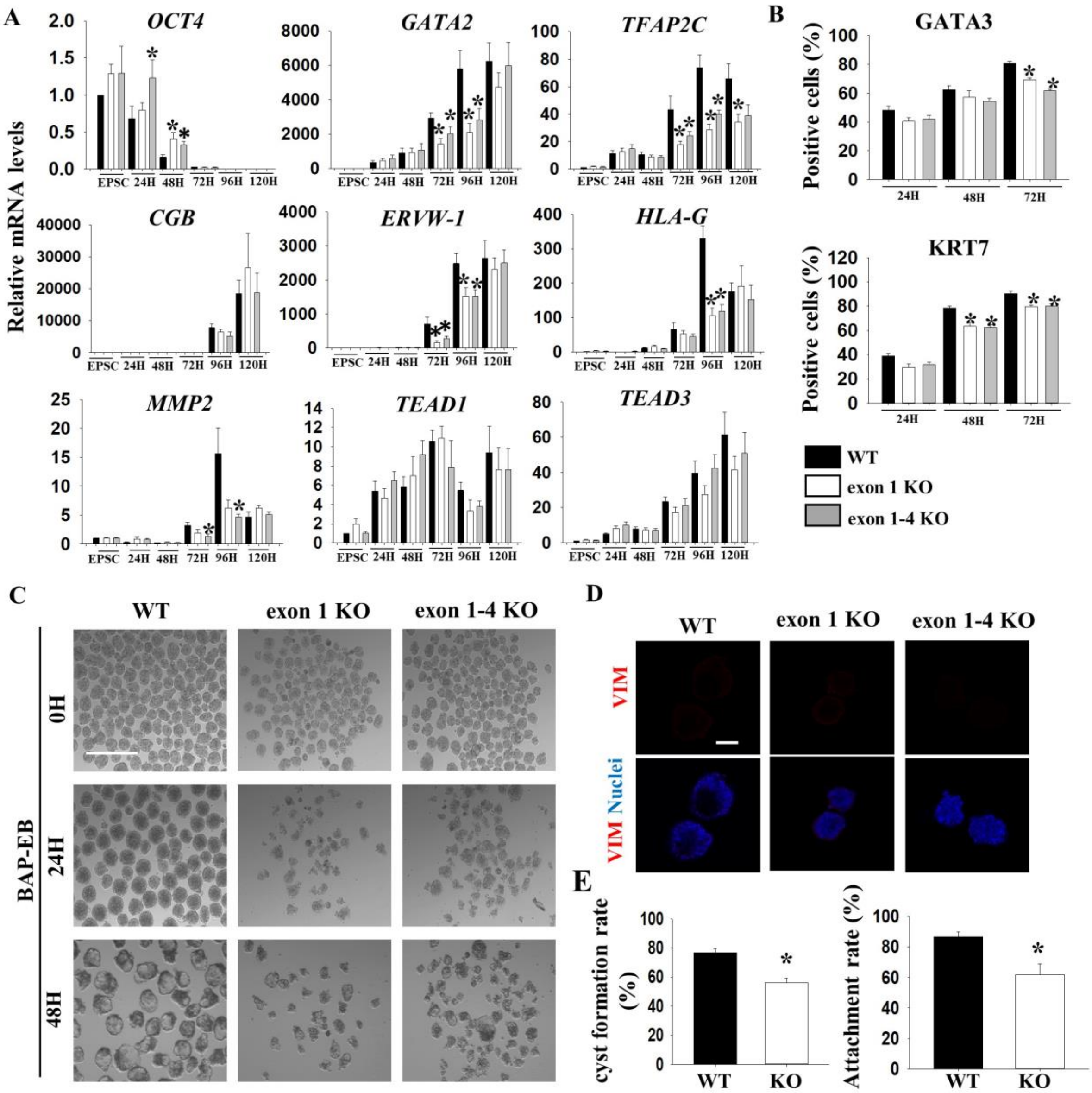

Fig. S9. YAP1 knockout inhibited trophoblast spheroid formation from hEPSC

(**A**)**:** RT-qPCR analysis of *OCT4*, *GATA2*, *TFAP2C*, *CGB*, *ERVW-1*, *HLA-G*, *MMP2*, *TEAD1* and *TEAD3* for trophoblast differentiation from hEPSC-em-4 wild-type and *YAP1* knockout cells at exon 1 and from exon 1-4. *: $p<0.05$ comparing with wild-type; t-test; n=5. (**B**)**:** FACS analysis showing proportion of GATA3- and KRT7-positive cells after trophoblast differentiation from hEPSC-em-4 wild-type and *YAP1* knockout cells at exon 1 and from exon 1-4. *: $p<0.05$ comparing with wild-type; t-test; n=5. **(C):** Representative figures showing BAP-EB differentiated from hEPSC-em-4 wild-type and *YAP1* knockout cells at exon 1 and from exon 1-4 at different time points. Scale bar: 100μm. (**D**)**:** Immunofluorescent staining of VIM in 48H BAP-EB derived from hEPSC-em-4 wild-type and *YAP1* knockout cells at exon 1 and from exon 1-4. The nuclei were stained with Hoest33258. Scale bar: 100μm. (**E**)**:**

Proportion of cystic BAP-EB formation (left) and the endometrial Ishikawa cell line attachment rates (right) between hEPSC-em-4 wild-type and *YAP1* knockout cells at exon 4. *: $p<0.05$ comparing with wild-type; t-test; n=3.
